# Evidence of inequities experienced by the rare disease community with respect to receipt of a diagnosis and access to services: a scoping review of UK and international evidence

**DOI:** 10.1186/s13023-025-03818-w

**Published:** 2025-06-12

**Authors:** Simon Briscoe, Clara Martin Pintado, Katy Sutcliffe, G. J. Melendez-Torres, Ruth Garside, Hassanat M. Lawal, Noreen Orr, Liz Shaw, Jo Thompson Coon

**Affiliations:** 1 University of Exeter Medical School, University of Exeter, Exeter, UK; 2 EPPI Centre, UCL Social Research Institute, University College London, London, UK

## Abstract

**Background:**

People with a rare disease find it difficult to obtain a diagnosis and access appropriate services. Evidence suggests that this can lead to health inequity amongst the rare disease community, i.e. systemic, unfair and avoidable differences in health opportunities and outcomes. This scoping review aims to identify and describe evidence on health inequities experienced by the rare disease community with regards to receipt of a diagnosis and access to health and social care services.

**Methods:**

We searched ASSIA, CINAHL, Embase, HMIC, MEDLINE and Social Policy and Practice for relevant studies. Studies were double screened at title and abstract and full-text using pre-specified inclusion criteria. As this research was commissioned by the UK National Institute for Health and Care Research Policy Research Programme, primary studies were limited to UK settings. These were supplemented with international systematic reviews. We also applied a 2010 date limit. Relevant data were extracted and presented narratively and tabulated.

**Results:**

One hundred thirty-six studies met the inclusion criteria, including 96 primary studies and 40 systematic reviews. The most frequently occurring rare diseases were motor neurone disease, cystic fibrosis and sickle cell disease. Seventeen types of inequity were identified: delayed diagnosis, lack of knowledge amongst clinicians, lack of information provision, limited services provision (across six different services), limited services for undiagnosed conditions, lack of care co-ordination; in addition, inequity was identified relating to place of residence, race/ethnicity, gender, socioeconomic status, age and disability.

**Conclusion:**

This review has drawn attention to experiences of the rare disease community with respect to receipt of a diagnosis and access to services which are different to experiences in the general population, and within the rare disease community itself. Some of these experiences are clearly attributable to factors which are unfair, avoidable and systemic, particularly those which relate to specific groups in the rare disease community. Experiences relating to delayed diagnosis, lack of knowledge, information, care co-ordination and access to various services, also appeared to indicate inequity. These issues are less likely to be encountered with respect to more common diseases experienced in the general population.

**Supplementary Information:**

The online version contains supplementary material available at 10.1186/s13023-025-03818-w.

## Background

Rare diseases are diseases which affect fewer than one in 2000 people [1]. Although rare diseases are individually rare, including some which are documented to affect only a handful of people worldwide, they are collectively common, with around 3,500,000 people in the United Kingdom (UK) (c. 5% of the population) affected by one of 7000 documented rare diseases [2]. People with a rare disease (PwRD) can find it difficult to access appropriate care [3]. A recent report found that, in the UK, this can arise due to factors such as limited knowledge of rare diseases amongst health care professionals (HCPs), poorly coordinated care, and scarcity of specialist centres for some conditions [4]. PwRD can also experience delayed diagnosis, leading to months or years spent with deteriorating health without receiving appropriate treatment [4]. These barriers to accessing care are likely to lead to worse health outcomes for PwRD than in the general population who do not encounter these barriers. They may also lead to differences in health outcome within the rare disease community, e.g. where resources are unevenly distributed regionally [3].

Differences in health outcomes which are due to wider determinants of health (i.e. non-medical factors such as ethnicity, gender and place of residence) are important to address in order to ensure that all people have equal access to appropriate care [5]. Where people do not have equal access to appropriate care this can lead to health inequity, i.e. systemic, avoidable and unfair differences in health outcomes between populations or population subgroups [6]. The England Rare Disease Action Plans are committed to addressing health inequities associated with rare diseases [2, 7, 8]. This builds on the UK Rare Diseases Framework, which commits to addressing health inequities [1].

In particular, the England 2023 Rare Disease Action Plan commits to gathering the evidence needed to evaluate whether rare diseases should be incorporated into the PLUS category of NHS England’s Core20PLUS5 framework, enabling integrated care systems (ICSs) to develop targeted actions to reduce inequalities. The Core20PLUS5 framework aims to support ICSs to reduce health inequities for people with complex and long-term conditions at a local and national level. Core20 refers to the most deprived 20% of the national population as identified by the national Index of Multiple Deprivation. The PLUS category includes population groups that are likely to experience poorer than average health opportunities and outcomes, such as ethnic minorities and people with a learning disability; 5 denotes five clinical areas requiring improvement: maternity, severe mental illness, chronic respiratory disease, early cancer diagnosis and hypertension and lipid management [9].

Although there are well documented examples of health inequity amongst PwRD, there is limited overall understanding of the extent of the evidence [10]. In response to a request from the UK National Institute for Health and Care Research (NIHR) Policy Research Programme to inform the UK Rare Disease Action Plan, this review aims to identify and summarise evidence on health inequities experienced within the rare disease community, or between the rare disease community and general population, with regards to receipt of a diagnosis and access to health and social care services. In particular, the review seeks to draw out findings relevant to the UK context to meet the needs of the review’s UK-based commissioner.

## Methods

**Fig. 1:**
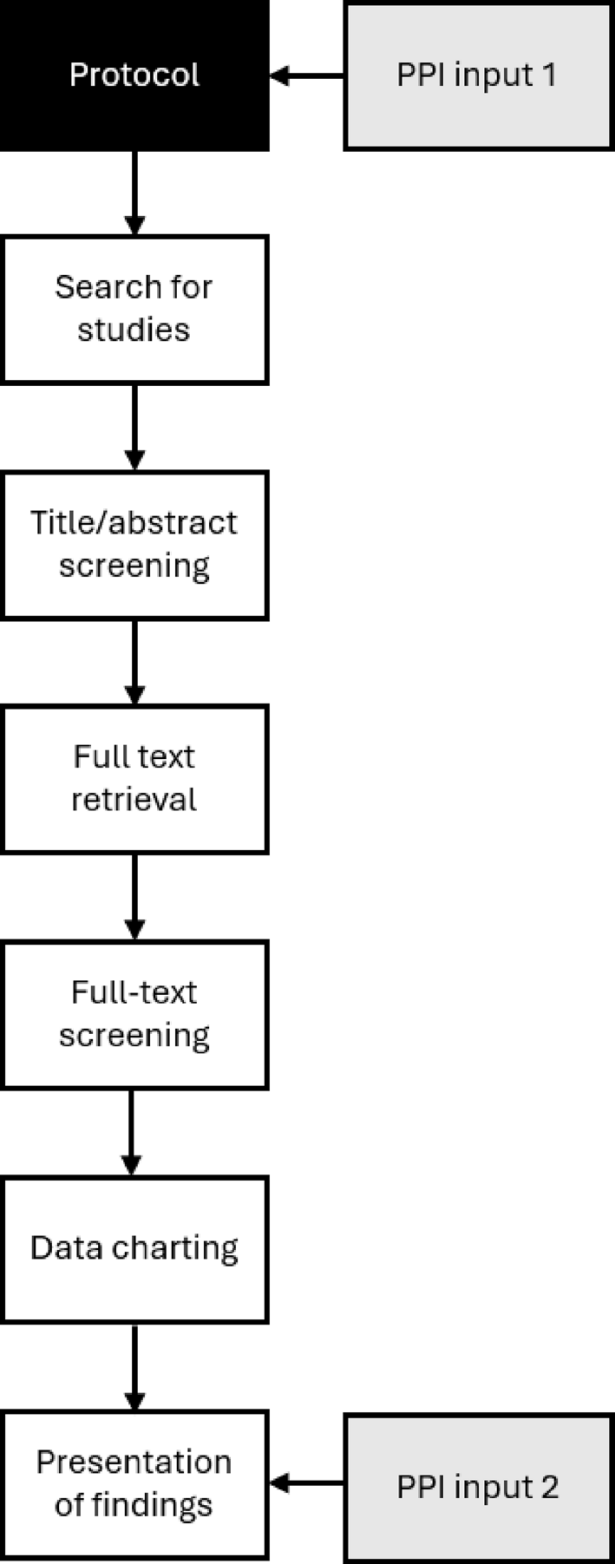
Flow diagram of scoping review methods

We followed established guidance on conducting and reporting scoping reviews [11, 12]. A protocol was registered prosectively [13]. A flow diagram of the methods used is presented in Fig. 1.

### Searches for studies and study selection

We searched the following bibliographic databases for relevant studies in February 2024: ASSIA (via ProQuest), CINAHL (via EBSCO), Embase, HMIC, MEDLINE and Social Policy and Practice (all via Ovid). The bibliographic database searches combined generic search terms for rare or genetic diseases, with search terms which describe types of potential inequity. We used generic terms for rare and genetic diseases as it was deemed impractical to search for all documented rare diseases individually. The search terms for rare diseases were derived from the UK Rare Disease Landscape search protocol [14]. Inequity search terms were derived from published search filters [15, 16], and supplemented with search terms which describe potential inequities identified from background reading, relating to knowledge, information, communication and literacy of rare diseases amongst HCPs and PwRD.

The final search strategy was developed and tested using a pre-identified set of relevant studies to ascertain that it retrieved all known relevant studies. This helped to ascertain that we did not miss studies by using generic terminology for rare and genetic diseases, or the included equity search terms. In particular, the test set of studies helped us to refine the equity search terms by informing the use of search terms for inequities specific to the rare disease community (e.g. lack of knowledge or information) which were not specified in the pre-designed search filters, allowing us to omit equity related search terms which retrieved a high number of studies which were not relevant. The bibliographic database searches are reproduced in Supplementary File 1.

We also checked the reference lists and carried out forward citation searches of relevant studies, inspected the journal contents pages of the Orphanet Journal of Rare Diseases, searched relevant websites, inspected the included studies of relevant systematic reviews (SRs), and asked experts to recommend studies. A full list of websites searches is listed in Supplementary File 1.

Studies were selected for inclusion if they reported experiences of inequity relating to receipt of a diagnosis or access to services for PwRD or carers of PwRD. Rare diseases were defined as diseases affecting fewer than one in 2000 people, with reference to the Orphanet website (https://www.orpha.net/) for confirming their status as rare diseases [1]. Rare cancers and infectious diseases were excluded following a request from the commissioner to keep the included diseases consistent with those in the Rare Disease Research Landscape project [17]. The context of inequity could include primary, secondary, tertiary and social care services. We included only UK primary studies to meet the specified objective of drawing out findings relevant to the UK context. We supplemented this with SRs which included at least one study from a World Bank high income country to provide wider context from comparable international literature. A 2010 date-limit was applied at the request of the commissioner to capture studies which are relevant to the contemporary context. Owing to the small amount of comparative data on inequities identified, non-comparative data on the experiences of PwRD relating to inequity were included and framed as *indicating* inequity. Non-comparative data included experiences of receipt of a diagnosis or access to services which were unlikely to be experienced outside of the rare community (e.g. lack of knowledge amongst HCPs about rare diseases) but were not explicitly comparative, e.g. qualitative interviews on the experiences of PwRD.

The titles and abstract of all identified studies were inspected for relevance using EPPI-Reviewer 6.0 (EPPI Centre Software, Social Science Research Unit, Institute of Education, University of London) by two independent reviewers. Disagreements were resolved through discussion, sometimes with input from a third reviewer or consultation with the commissioner. The full-texts of titles and abstracts meeting the inclusion criteria were retrieved and screened in the same way.

### Charting the data

Key characteristics of primary studies and SRs which met our inclusion criteria were extracted into separate data extraction forms. Data on inequity was coded as to whether it was relevant to receipt of a diagnosis or access to services, and whether the type of inequity was shared across the rare disease community, or related to specific groups as described in the PROGRESS + framework [18]. PROGRESS + is a framework which sets out characteristics which stratify health opportunities and outcomes, which was used as a guide to identifying inequities [18]. For the inequities which are shared across PwRD, we differentiated between inequities between the rare disease community and general population, and inequities within the rare disease community, i.e. where data compared experiences for different types of rare diseases.

### Patient and public involvement (PPI)

The protocol was discussed with the standing PPI group at the Exeter PRP Evidence Review Facility, PERSPEX [13]. We also convened a PPI group of four people with lived experience of rare diseases. This group met on 17th July 2024 to discuss preliminary findings of our review.

### Presentation of findings

The key characteristics of the identified studies which met our inclusion criteria are presented in tabulated format and described narratively. Primary studies and SRs are presented separately. Data on inequities are described narratively in two parts: first, inequities with respect to receipt of a diagnosis; secondly, inequities with respect to access to services. Within these sections, we summarise the data as these relate to inequities which are shared across the rare disease community (drawing out where there is comparative data within the rare disease community); and specific groups within the rare disease community, with reference to PROGRESS + [18]. Content analysis was undertaken to draw out relevant detail in the identified studies.

## Findings

### Study identification

**Fig. 2:**
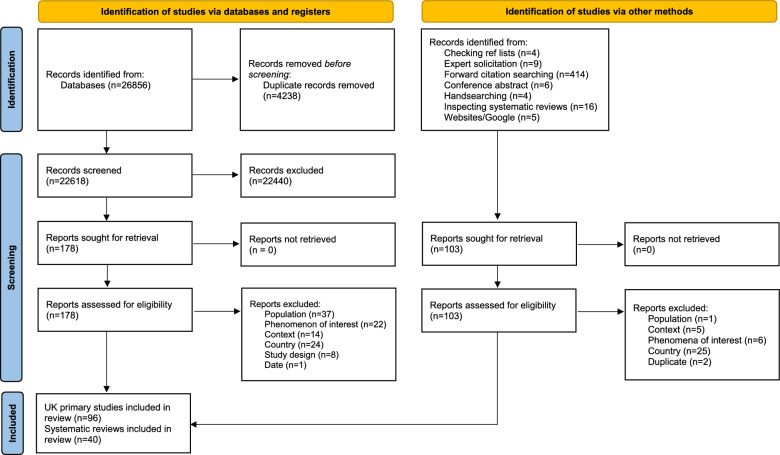
PRISMA flow diagram

In total we identified 136 relevant studies, including 96 UK primary studies [4, 19–113] and 40 SRs [114–153]. of these, 45 primary studies [19, 20, 25, 26, 33, 34, 38–40, 42–44, 46–49, 51–54, 57, 58, 60, 61, 66–68, 72, 75, 76, 78, 80, 82, 86–90, 94–97, 100, 105, 113] and 17 SRs were relevant to receipt of a diagnosis [114–116, 118, 122, 127, 129, 131, 136, 138–140, 142, 147, 150–152], and 76 primary studies [4, 19, 21–32, 35–37, 40–42, 44, 45, 47–52, 55, 56, 59, 60, 62–79, 81, 83–85, 87, 88, 91–94, 96–99, 101–112] and 37 SRs were relevant to access to health and social care services [114, 116–128, 130–142, 144–153]. The study identification process is documented in the PRISMA flow diagram in Fig. 2 [12].

### Key characteristics of included studies

The included primary studies and SRs comprise 122 journal articles, 11 grey literature reports [4, 47, 48, 58, 67, 75–78, 101, 113] and three theses [94, 95, 132]. The key characteristics of included primary studies and SRs are presented in full in Table S1 and Table S2 respectively in Supplementary File 2.

Qualitative studies were the most frequently occurring primary study design (n = 50, 52%) [4, 19–21, 23, 24, 26, 27, 30, 33, 37–39, 41–45, 47–49, 51–53, 55–57, 59, 60, 62, 68, 69, 71, 72, 81–83, 85, 87, 88, 90–92, 94–96, 101, 103, 107, 110] including two case studies with qualitative data [20, 48]. Twenty-one (22%) primary studies were quantitative studies [22, 29, 32, 34, 35, 46, 50, 61, 63, 74, 76, 77, 86, 93, 98–100, 102, 106, 108, 113] and 25 (26%) were mixed methods studies which included both qualitative and quantitative data [25, 28, 31, 36, 40, 54, 58, 64–67, 70, 73, 75, 78–80, 84, 89, 97, 104, 105, 109, 111, 112]. The majority of quantitative data in the quantitative studies were derived from survey or questionnaire data (n = 16, 76%) [23, 29, 32, 46, 61, 63, 74, 76, 77, 93, 98–100, 102, 106, 108]. Three studies used hospital records data [34, 35, 50], one study used a pre-existing genetic dataset [113], and one was a prospective observational study [86]. Similarly, survey and questionnaire data was the most commonly reported quantitative data in the mixed methods studies (n = 23, 92%) [25, 28, 31, 36, 40, 54, 64–67, 70, 73, 75, 78–80, 89, 97, 104, 105, 109, 111, 112]. Two mixed methods studies used quantitative medical records data [58, 84]. Of the SRs, eight (20%) included solely qualitative studies [115, 124, 126, 127, 131, 135, 143, 151, 152], two (5%) included solely quantitative studies [119, 133] and 30 (75%) included multiple study designs including qualitative, quantitative and mixed methods studies [114–118, 120–123, 125, 128–130, 132, 134, 136–142, 144–150, 153]. The median number of studies included in the SRs was 24.5 (range 6–59).

The number of rare diseases reported per primary study and SR ranged from one to > 450 [75]. Although most studies and SRs reported which rare diseases were included in their findings, eight studies did not provide an exhaustive report (see Table S3 in Supplementary File 3) and there were also studies which reported types of rare disease without breaking these down into subtypes, e.g. studies which reported MND but not which subtypes of MND. The most frequently reported rare diseases are presented in Table 1. These include MND, sickle cell disease (SCD) and cystic fibrosis (CF) as the most frequently reported rare diseases across both primary studies and SRs.

**Table 1:** Most frequently reported rare disease across primary studies and SRs

Primary studies	Systematic reviews
*Diseases reported in* ≥ *10 studies (n* = *number of studies)*	
Motor Neuron Disease (MND) (n = 14)	MND (n = 12)
	SCD (n = 10)^†^
*Diseases reported in* ≥ *5 studies (n)*	
Sickle cell disease (SCD) (n = 9)	CF (n = 7)
Cystic fibrosis (CF) (n = 7)^*^	Duchenne muscular dystrophy (DMD) (n = 6)
Inherited bleeding disorders (n = 6)	Inherited bleeding disorders (n = 5)
Ataxia (n = 5)	

^*^Includes one study on CF diabetes

^†^includes one study on SCD anaemia

Table 2 presents a breakdown of rare diseases within studies which include multiple subtypes of a rare disease (e.g. MND) or categories of rare disease (e.g. inherited bleeding disorders).

**Table 2:** Primary studies and systematic reviews which include multiple types of rare disease which are either subtypes or categorised together

Disease	Primary studies, n (systematic reviews, n)	Specific types of disease: primary studies, n (systematic reviews, n)*
*Rare disease with subtypes*		
Motor neurone disease	14 (12)	Amyotrophic lateral sclerosis (bulbar/pseudobulbar onset), 4(1); amyotrophic lateral sclerosis (limb onset), 4(1); amyotrophic lateral sclerosis (not specified), 2(6); brachial amyotrophic diplegia, 2(0); familial motor neuron disease/amyotrophic lateral sclerosis, 2(0); primary lateral sclerosis, 4(1); progressive bulbar palsy, 4(1); progressive muscular atrophy, 4(1); respiratory onset, 0(1); spinal muscular atrophy (not specified), 2(2); spinal muscular atrophy (type 1), (2); spinal muscular atrophy (type 2), (2); spinal onset, 0(1); not specified, 8(2);
Hypermobile Ehlers-Danlos syndrome	3 (1)	Ehlers-Danlos hypermobility (type II), 0(1); Ehlers-Danlos Syndrome (type III), 2(1); fibromyalgia, 1(0); not specified, 2(0)
Epidermolysis bullosa	1 (2)	Dystrophic Epidermolysis Bullosa, 1(0); Epidermolysis bullosa simplex, 1(0); junctional Epidermolysis Bullosa, 1(0); not specified 0(2)
Ataxia	5 (0)	Fragile X, 1(0); Friedreich's ataxia, 2(0); idiopathic cerebral ataxia, 1(0); spinocerebellar ataxia (general), 1(0); spinocerebellar ataxia (type 1), 1(0); spinocerebellar ataxia (type 2), 1(0); spinocerebellar ataxia (type 6), 1(0); spinocerebellar ataxia (type 7), 1(0); spinocerebellar ataxia (type 8), 1(0); not specified, 2(0);
*Rare diseases categorized together*		
Childhood dementias	0 (1)	Barth syndrome; CLN3 disease; complex I deficiency; complex III deficiency; cytochrome oxidase deficiency; D-bifunctional protein deficiency; genetically determined leukoencephalopathies; Kearns-Sayre syndrome; lactic acid-anaemia and stroke-like episodes (MELAS); Leigh disease; metachromatic leukodystrophy; mitochondrial encephalomyopathy, Mucopolysaccharidosis (type I, II and III); mucopolysaccharidosis (type I, Hurler syndrome); mucopolysaccharidosis (type II, Hunter syndrome); mucopolysaccharidosis (type III, Sanfilippo syndrome); mucopolysaccharidosis (type III subtype A, subtype B, or subtype C); multiple complex deficiency; NARP syndrome; Pyruvate dehydrogenase complex deficiency; Rett syndrome; X-linked adrenoleukodystrophy; Zellweger spectrum disorders
Inherited bleeding disorders	6 (5)	Bernard-Soulier syndrome, 1(1); diagnosed bleeding disorder (unspecified), 1(1); factor V deficiency, 1(0); factor VII deficiency, 1(0); factor X deficiency, 1(0); factor XI deficiency, 1(0); factor XIII deficiency, 1(0); Glanzmann’s disease, 3(0); haemophilia A/B, 4(5); haemophilia carriers, 0(2); immune thrombocytopenia, 1(0); other factor deficiencies, 1(0); platelet disorders-general, 3(1); platelet disorder-inherited thrombocytopenia, 1(0); thrombotic thrombocytopenia purpura, 1(0); Von Willebrand’s, 3(1); not specified, 1(2)
Genetic diseases†	2 (0)	NR
Rare neurodegenerative conditions	1 (0)	Charcot Marie Tooth disease; dominantly inherited ataxia; Huntington’s disease; motor neuron disease; multiple system atrophy; post-polio syndrome; progressive supranuclear palsy;
Non-cancer related rare disease	0 (1)	Huntington Disease; Telangiectasia; unspecified genetic diseases
Rare genetic intellectual disability syndromes	1 (0)	Angelman syndrome; Cornelia de Lange syndrome; Cri du Chat syndrome
Rare epilepsy-related disorders and intellectual disabilities	0 (1)	Dravet syndrome; Dravet syndrome/Lennox-Gastaut syndrome; tuberous sclerosis complex
Retinal dystrophies	1 (0)	Choroideremia; cone dystrophy; cone-rod dystrophy; Leber’s congenital amaurosis; Retinitis pigmentosa; retinoschisis; Sorsby fundus dystrophy; unspecified retinal or macular dystrophy
Previously undiagnosed developmental disorders	1 (0)	Abnormal growth parameters dysmorphic features; congenital anomalies; genetic disorders with a significant impact for which the molecular basis was unknown; Neurodevelopmental disorders; unusual behavioural phenotypes;

NR = not reported; *If not specified, subtype appears in only one study; † = these could include non-rare as well as rare condition

## Inequities experienced by the rare disease community

We identified 17 types of inequity across the 136 included studies. Of these, 11 were shared across the rare disease community and six related to specific groups within the rare disease community, as described in the PROGRESS + framework [18]. The different types of inequity identified within each of these categories are detailed in Tables 3 and 4 respectively.

**Table 3:** Types of inequity shared across the rare disease community

Type of inequity	Description reported by PwRD and their carers
Delayed diagnosis	Experiencing delays to receiving a diagnosis
Lack of knowledge	Lack of knowledge amongst health care professionals when seeking or receiving a diagnosis of a rare disease and when accessing services
Lack of information	Health care professionals did not provide or signpost to sufficient information about their disease when seeking or receiving a diagnosis of a rare disease and when accessing services
Limited service provision (comprising six types of inequity)	Challenges with accessing appropriate services, often related to limited services provision but also more specific challenges within different types of services. These included mental health services, emergency services, dentistry services, specialist services, social care services and services in general where these were not specified
Limited services for undiagnosed conditions	Specific challenges among those with symptoms of a rare disease around accessing services when they do not have a diagnosis of a rare condition
Lack of care co-ordination	Experiencing a lack of care co-ordination which adversely affects the receipt of a diagnosis and accessing services after a diagnosis is received

**Table 4:** Types of inequity identified with reference to PROGRESS +

Type of inequity	Description reported by PwRD and their carers
Place of residence	Geographic location is relevant to the treatment and support which they receive
Race/ethnicity	Ethnicity is relevant to the treatment and support which they receive
Gender	Gender is relevant to the treatment and support which they receive
Socioeconomic status	Socioeconomic status is relevant to the treatment and support which they receive
Age	Age is relevant to the treatment and support which they receive
Disability	Disability is relevant to the treatment and support which they receive

## Inequities with respect to receipt of a diagnosis

Twelve types of inequity were identified with respect to receipt of a diagnosis. Of these, six were experienced across the rare disease community, including four which included comparative data from within the rare disease community. An additional six types of inequity were experienced by subgroups within the rare disease community described in the PROGRESS + framework [18]. These types of inequity were identified in 45 primary studies [19, 20, 25, 26, 33, 34, 38–40, 42–44, 46–49, 51–54, 57, 58, 60, 61, 66–68, 72, 75, 76, 78, 80, 82, 86–90, 94–97, 100, 105, 113] and 17 SRs [114–116, 118, 122, 127, 129, 131, 136, 138–140, 142, 147, 150–152].

### Inequities experienced across the rare disease community with respect to receipt of a diagnosis

**Fig. 3:**
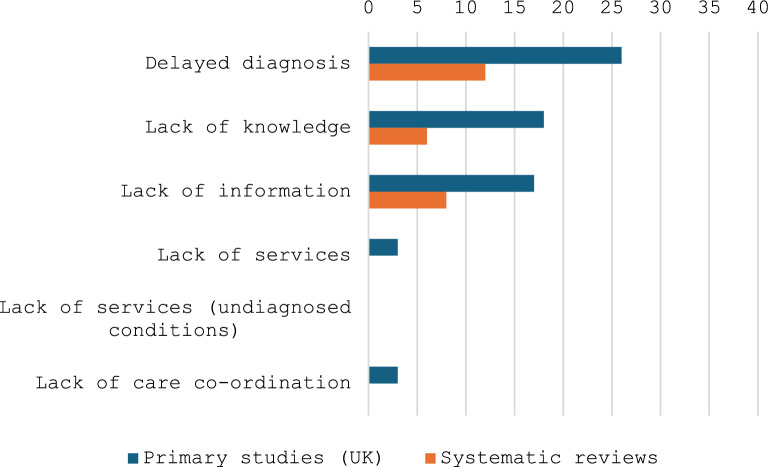
Inequities shared across the rare disease community with respect to diagnosis

An overview of the numbers of studies indicating different types of inequity across the rare disease community with respect to receipt of a diagnosis is presented in Fig. 3.

#### Delayed diagnosis

Data indicated that delays to the diagnosis of rare diseases listed in Table 5 may lead to inequity between PwRD and the general population, who do not routinely experience similar delays to diagnosis (Primary studies [PS], n = 26 [19, 20, 25, 26, 38, 42, 43, 46–48, 51–53, 58, 60, 67, 68, 72, 75, 76, 78, 80, 82, 88, 95, 96]; Systematic reviews [SR], n = 12 [70, 114, 116, 118, 122, 127, 131, 138, 139, 142, 151, 152]). The relevant data in these studies were predominantly qualitative data or non-comparative survey data, with one SR reporting comparative quantitative data [139].

**Table 5:** Rare diseases in UK primary studies and SRs relating to delayed diagnosis

UK primary studies (n)	Systematic reviews (n)
Alstrom syndrome (1) [48]	Childhood dementias (1) [138]
ANCA-associated Vasculitis (1) [58]	Fragile X syndrome (1) [131]
Ataxia and progressive ataxia (2) [42, 43]	MND (3) [116, 122, 127, 142]
Bardet Biedl Syndrome (1) [58]	Frontotemporal lobar degeneration (1) [139]
Cavernoma (1) [48]	Ehlers–Danlos syndrome (1) [114]
Chronic intestinal pseudo-obstruction (1) [48]	Primary ciliary dyskinesia (1) [118]
CF (1) [95]	Multiple rare diseases (3: Mc Mullan 2022; von der Lippe 2017; von der Lippe 2022) [70, 151, 152]
Deletion on chromosome 4q (1) [48]	–
Desmoid fibromatosis (1) [60]	–
Dystonia (1) [72]	–
Ehlers-Danlos syndrome (3) [25, 26, 48]	–
Guillain–Barre Syndrome (1) [19]	–
Hereditary spastic paraparesis (1) [52]	–
MND (4) [53, 80, 82, 88]	–
Multiple System Atrophy (MSA) (2) [76, 78]	–
Rare genetic intellectual disabilities (1) [51]	–
Retinal dystrophies (1) [38]	–
SCD (1) [48]	–
Trimethylaminuria (1) [46]	–
Tuberous sclerosis complex (2) [58, 68]	–
Tumour Necrosis Factor Receptor Associated Periodic Syndrome (TRAPS) (1) [20]	–
Multiple rare diseases (4: Franklish 2022; Limb 2010; Muir 2016; Simpson 2021) [47, 67, 75, 96]	–

Data in primary studies indicated the perception amongst PwRD and their carers that clinicians often lack the requisite knowledge to make a timely diagnosis [26, 51–53]. Misdiagnosis could also delay accurate diagnosis [19, 20, 60, 75, 78]. PwRD and carers also reported delays to diagnosis arising from clinicians’ reluctance to “label” someone with a rare disease [25, 42, 43, 47, 60, 68, 72, 75, 95], and the need for many referrals and long wait times for appointments [38, 47, 48, 58, 60, 82]. Data in SRs indicated similar experiences, including quantitative data which compared the time to diagnosis for people with frontotemporal lobar degeneration (59.2 months) and Alzheimer’s disease (39.1 months), as a comparison between time to diagnosis of a rare disease and similar disease experienced in the general population [139]. Misdiagnosis was reported as a recurrent issue for those affected by frontotemporal lobar degeneration, with one study reporting that out of 19 patients with frontotemporal lobar degeneration, 18 were initially misdiagnosed with depression, manic depression, psychosis or dementia [139]. In some cases, this lack of knowledge, combined with the lack of apparent and visible symptoms in some rare diseases, could lead clinicians to accuse patients of fabricating illnesses and dismissing their symptoms as unimportant or non-existent [114, 122, 131, 142]. Patients and carers also reported not being referred to the appropriate specialists for diagnosis, or waiting long times until they were eventually referred [114, 118, 138, 142].

Data also indicated that delays to the diagnosis of a rare disease may lead to inequity within the rare disease community. Quantitative survey data showed that people with MND experience a wide range of time to diagnosis (median 398 days; range 35–3348 days) [80].

#### Lack of knowledge

Data indicated that clinicians’ limited knowledge of rare diseases listed in Table 6 may lead to inequity between PwRD and the general population in the context of diagnosis (PS, n = 18 [19, 25, 26, 33, 38, 42, 46, 47, 51, 53, 67, 72, 75, 82, 88, 89, 97, 105]; SR, n = 6 [114, 129, 131, 138, 151, 152]) The relevant data in these studies comprised of qualitative and non-comparative survey data.

**Table 6:** Rare diseases in UK primary studies and SRs relating to lack of knowledge and diagnosis

UK primary studies (n)	Systematic reviews (n)
Adrenal Insufficiency (1) [97]	Childhood dementias (1) [138]
Ataxia (1) [42]	Ehlers–Danlos syndrome (1) [114]
CF (1) [42]	Fragile X syndrome (1) [131]
Ehlers–Danlos syndrome (2) [25, 26]	MND (1) [129]
MND (2) [82, 88]	Multiple rare diseases (2: von der Lippe 2017; von der Lippe 2022) [151, 152]
Polyneuropathy Organomegaly Endocrinopathy Monoclonal gammopathy Skin changes (POEMS) syndrome (1) [105]	–
Rare genetic intellectual disabilities (1) [51]	–
SCD (2) [42]	–
Trimethylaminuria (1) [46]	–
Multiple rare disease (4: Franklish 2022; Limb 2010; Muir 2016; Peter 2022) [47, 67, 75, 89]	–

Primary studies indicated the perception amongst PwRD and carers that clinicians had a general lack of awareness of symptoms of rare diseases [26, 33, 42, 46, 47, 51, 57, 67, 75, 88, 97, 105], and dismissive attitudes towards symptoms [25, 67, 82, 97], which sometimes led to delays to diagnosis. Dismissive attitudes included dismissiveness towards the existence of hypermobile syndromes [25], dismissal from GP consultation without further investigation of symptoms of multiple rare diseases [67, 82], and feeling the need to “fight” for clinician acknowledgement of symptoms of adrenal insufficiency [97]. Data in SRs indicated similar experiences, including dismissive attitudes towards symptoms, particularly towards the existence of hypermobile syndromes [114]. Even when a diagnosis was achieved, PwRD and their carers reported dissatisfaction with the communication and accuracy of the diagnosis [129], and that clinicians were unwilling to address their limited knowledge of a rare condition through learning more about it, which affected PwRD ability to access appropriate services [138].

Data also indicated that clinicians’ limited knowledge about rare diseases when making a diagnosis may lead to inequity within the rare disease community. Quantitative survey data showed that people with MND seen at a specialist clinic were more likely to be satisfied with their experience of diagnosis than people not seen in an MND specialist clinic [80].

#### Lack of information

Data indicated that PwRD and their carers may receive less information about rare diseases listed in Table 7 at the point of diagnosis compared to diseases experienced in the general population (PS, n = 17 [19, 39, 40, 47, 54, 57, 61, 67, 75, 76, 78, 80, 82, 87–89, 95]; SR, n = 8 [115, 116, 127, 131, 136, 138, 140, 150]). The relevant data in these studies comprised of qualitative and non-comparative survey data.

**Table 7:** Rare diseases in UK primary studies and SRs relating to lack of information and diagnosis

UK primary studies (n)	Systematic reviews (n)
Acromegaly (1) [87]	Childhood dementias (1) [138]
CF (1) [95]	Fragile X syndrome (1) [131]
Guillain–Barre Syndrome (1) [19]	MND including one specifically on ALS (4) [115, 116, 127, 140]
Lysomal acid lipase deficiency (1) [57]	MS (1) [115]
MND (4) [54, 80, 82, 88]	Multiple rare diseases (2: McMullan 2022; Tsitsani 2023) [136, 150]
MSA (1) [76, 78]	–
Multiple rare diseases (5: Costa 2022; Crowe 2019; Franklish 2022; Hytiris 2021; Limb 2010; Muir 2016; Peter 2022) [39, 40, 47, 61, 67, 75, 89]	–

Data in primary studies mainly focused on information needs immediately after a diagnosis. Specifically, data indicated that PwRD and carers have unanswered questions about their diagnosis [39], and specific information needs around treatment options, services and care pathways [40, 80]. PwRD also reported challenging experiences trying to find information on the internet after a diagnosis [57]. Similarly, most SRs reported a lack of information immediately after a diagnosis. PwRD felt that clinicians were unable to signpost them to reliable information sources [115] or to adequate support, such as rare disease family groups, psychologists and social services [150]. PwRD and carers also reported specific information needs relating to treatment plans [115, 116], prognosis [115, 116] and caring skills [140]. One SR reported a lack of information during the diagnostic process, with parents reporting insufficient information around the diagnostic tests their child was undergoing [138].

Data also indicated that PwRD and their carers may receive differing amounts of information about their disease at the point of diagnosis, which may lead to inequity within the rare disease community. Quantitative survey data showed that people who receive a diagnosis of MND in neurology clinics and their carers received less than half of the information about their diagnosis recommended in NICE guidance [80]. In comparison, for people who receive a diagnosis of MND in MND specialist centres there was greater, albeit not full, compliance with the information provision recommended in NICE guidance [80].

#### Lack of care co-ordination

Data indicated that PwRD and their carers experienced a lack of care co-ordination when receiving a diagnosis of rare diseases listed in Table 8, which may not be experienced by the general population who do not typically have similarly complex care needs (PS, n = 3) [25, 47, 54].

**Table 8:** Rare diseases in UK primary studies relating to care co-ordination and diagnosis

Rare diseases (studies, n)
Ehlers–Danlos syndrome (1) [25]
MND (1) [54]
Multiple rare diseases (1: Franklish 2022) [47]

Qualitative data indicated the perception that there was a lack of clarity around pathways to diagnosis, and confusion amongst both clinicians and PwRD about the different clinicians involved in the diagnostic pathway [25, 47, 54].

### Access to services (with respect to receipt of a diagnosis)

#### Specialist services

Data on specialist services indicated that PwRD and their carers experienced limited access to specialist services when seeking diagnostic tests (PS, n = 2) [47, 80] Data in one study showed that people who were diagnosed with MND in a specialist centre were more likely to report a high satisfaction rating than those not diagnosed at an MND specialist centre [80]. A contributing factor to this was a more frequently offered invitation for follow up discussion with a neurologist in specialist centres compared with outside of specialist centres [80]. This could lead to inequity both between the rare disease community and general population, and within the rare disease community. Similar data was reported with respect to experiences of people with ataxia [74, 106]. Also, PwRD perceived that primary care clinicians were reluctant to refer for them for diagnostic tests due to a “proprietorial” attitude towards them (“…as if they [i.e. the clinician] felt a sense of ownership and were afraid of losing control over the [patient’s] care”) [47]. These PwRD reported feeling the need to “fight” or “struggle” for access to services for a diagnosis [47].

#### Mental health services

Qualitative data in one study indicated that people with symptoms of a rare disease requested access to mental health services when seeking a diagnosis, but it was either not available or there were long waiting times [47]. This related to multiple rare diseases [47]. This potentially leads to inequity between the rare disease community and the general population. However, difficulty accessing mental health services is also reported in the general population [154].

### Inequities experienced by specific groups in the rare disease community with respect to receipt of a diagnosis

**Fig. 4:**
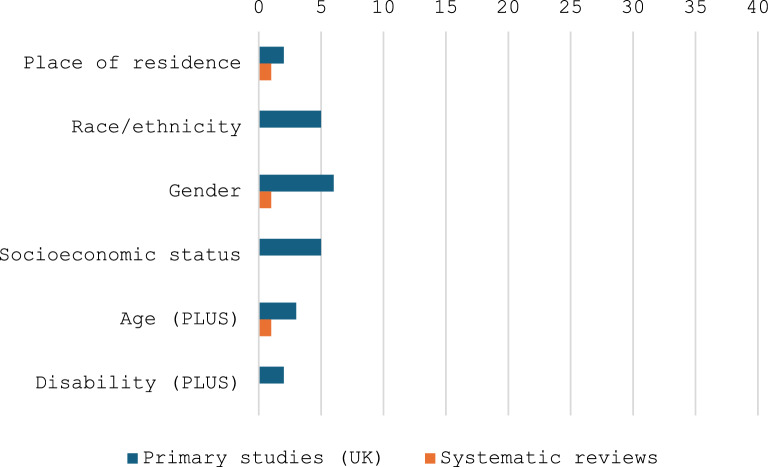
PROGRESS + inequities with respect to diagnosis

An overview of the numbers of studies indicating different types of inequity associated with specific groups within the rare disease community, as identified with reference to PRORGRESS +, with respect to receipt of a diagnosis is presented in Fig. 4 [18].

#### Place of residence

Data indicated that geographic location may impact on the experience of receipt of a rare disease diagnosis for PwRD and their carers (PS, n = 2 [47, 90]; SR, n = 1 [150]). Multiple rare disease were reported in primary studies [47, 90] and one SR [150]. The data indicated that PwRD face challenges in accessing services for diagnostic tests where this requires travelling to tertiary services which are far away from their home, including financial challenges with respect to cost of travel [47, 90, 150]. This may not be experienced in the general population who do not need access to specialist services, and access may vary within the rare disease community depending on how far they live from a specialist centre.

#### Race/ethnicity

Data indicated that the ethnicity of PwRD may impact on the experience of receipt of a rare disease diagnosis listed in Table 9 (PS, n = 5) [34, 47, 86, 94, 113].

**Table 9:** Rare diseases in UK primary studies relating to race/ethnicity and diagnosis

Rare disease (studies, n)
Developmental disorders (1) [113]
Fabry disease (1) [34]
Medium chain acyl-CoA dehydrogenase deficiency (1) [86]
Rare neurodegenerative conditions (1) [94]
Multiple rare diseases (1: Franklish 2022) [47]

Qualitative data in two primary studies indicated that ethnic minority patients with symptoms of a rare disease may receive different treatment to white British patients [47, 94]. One study reported the perception that ethnicity may be a factor in whether a patient is believed regarding their symptoms [47], and one study reported that language may be barrier to conveying symptoms to clinicians for ethnic minority patients [94]. Additionally, quantitative data in three UK primary studies showed that ethnic minority patients may be less likely to receive a diagnosis of a rare disease than white British patients [34, 86, 113]. This included Medium chain acyl-CoA dehydrogenase deficiency [86], Fabry disease [34], and multiple rare genomic diseases [113]. This outcome was explained in one study as due to limited ethnic minority representation in genetic datasets [113].

#### Gender

Data indicated that the gender or sex of PwRD or their carers may impact on the experience of receipt of a rare disease diagnosis listed in Table 10 (PS, n = 6 [25, 47, 49, 66, 100, 113]; SR, n = 1 [147]).

**Table 10:** Rare diseases in UK primary studies and SRs relating to gender and diagnosis

UK primary studies (n)	Systematic reviews (n)
Developmental disorders (1) [113]	Inherited bleeding disorders [147] (1)
Ehlers–Danlos syndrome (1) [25]	-
Fibrous dysplasia (1) [100]	-
McCune-Albright syndrome (1) [100]	-
Inherited bleeding disorders (1) [66]	-
Mayer-Rokitansky-Kuster-Hauser syndrome (1) [49]	-
Multiple rare diseases (1: Franklish 2022) [47]	-

Qualitative data in four primary studies indicated that women perceived dismissive attitudes towards their symptoms, or the symptoms of their children, prior to diagnosis [25, 47, 49, 66]. This included the attribution of concern about their child’s symptoms to mental ill health [25], and dismissal of their symptoms as within the bounds of normal menstrual health [66]. One SR similarly reported dismissive attitudes towards symptoms in female patients, leading to misdiagnosis and diagnostic delay [147].

Additionally, quantitative survey data in one primary study reported that women experience a long time to diagnosis of a rare disease, specifically, fibrous dysplasia and McCune-Albright syndrome [100]. Similarly, one SR reported that women experience a longer time to diagnosis than men for bleeding disorders (14.0 ± 16.6 years vs 8.1 ± 17.0 years; P < 0.001) [147]. Contrastingly, one primary study reported data that a diagnosis was less likely to be obtained for male patients (OR: 0.72, 95% CI: 0.67–0.79) [113].

#### Socioeconomic status

Data indicated that the financial status of PwRD or carers may impact on the experience of receipt of a rare disease diagnosis listed in Table 11 (PS, n = 4) [25, 47, 82, 88, 90].

**Table 11:** Rare diseases in UK primary studies relating to socioeconomic status and diagnosis

Rare diseases (studies, n)
Ehlers–Danlos syndrome (1) [25]
Genetic diseases (1) [90]
MND (2) [82, 88]
Multiple rare diseases (1: Franklish 2022) [47]

Qualitative data in primary studies indicated that people with symptoms of a rare disease or their carers sometimes used their financial resources to access diagnostic tests for a rare disease from private health care services [25, 82, 88, 90]. This was carried out due to delays to the receipt of a diagnosis from NHS service providers, even if this resulted in “significant personal cost” [88]. Within the NHS, patients were sometimes signposted to diagnostic services outside of their locality which they did not have sufficient funds to travel to [47].

#### Age

Data indicated that the age of PwRD may impact on the experience of receipt of a rare disease diagnosis listed in Table 12 (PS, n = 3 [25, 47, 100]; SR, n = 1 [139]).

**Table 12:** Rare diseases in UK primary studies and SRs relating to age and diagnosis

UK primary studies (n)	Systematic reviews (n)
Ehlers–Danlos syndrome (1) [25]	Frontotemporal lobar degeneration (1) [139]
Fibrous dysplasia (1) [100]	–
McCune-Albright syndrome (1) [100]	–
Multiple rare diseases (1: Franklish 2022) [47]	–

Qualitative data in primary studies reported that clinicians were reluctant to give a child a diagnostic label of Ehlers-Danlos syndrome [25], and that being a child could mean that clinicians are less trusting of what they say about their symptoms [47]. It was not clear whether reluctance to give a diagnosis of Ehlers-Danlos syndrome related to dismissive attitudes towards the condition, or to a diagnostic decision process of first excluding other possible conditions. Additionally, quantitative survey data in one UK primary study reported that people who experience symptoms of fibrous dysplasia or McCune-Albright syndrome at a young or older age can have a longer time to diagnosis than people who experience symptoms in between these age groups [100].

Quantitative data in one SR reported that a diagnosis of early onset dementia is often hindered due to lack of knowledge amongst health care professionals [139].

#### Disability

Data indicated that disabled people may have different experiences of receipt of a diagnosis to able-bodied people (PS, n = 2) [51, 94]. Rare diseases reported in these studies included rare genetic intellectual disabilities [51] and rare neurodegenerative conditions [94]. Qualitative data indicated the perception that symptoms of a rare disease were sometimes erroneously attributed to pre-existing disability [94], and that there was a prejudice against diagnosing intellectual disabilities, including rare genetic intellectual disabilities [51].

## Inequities with respect to access to services

Sixteen types of inequity were identified with respect to access to services. Of these, 10 were experienced across the rare disease community, including three which included comparative data from within the rare disease community. An additional six types of inequity were experienced by subgroups within the rare disease community described by the PROGRESS + framework [18]. These types of inequity were identified in 76 primary studies [4, 19, 21–32, 35–37, 40–42, 44, 45, 47–52, 55, 56, 59, 60, 62–79, 81, 83–85, 87, 88, 91–94, 96–99, 101–112] and 37 SRs [114, 116–128, 130–142, 144–153].

### Inequities across the rare disease community with respect to access to services

**Fig. 5:**
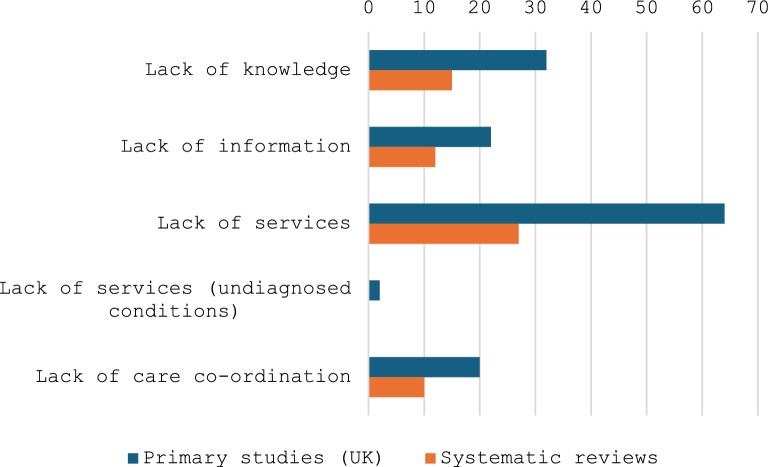
Inequities shared across the rare disease community with respect to access to services

An overview of the numbers of studies indicating inequities across the rare disease community with respect to access to services is presented in Fig. 5.

#### Lack of knowledge

Data indicated that clinicians’ limited knowledge about rare diseases listed in Table 13 may lead to inequity between PwRD and the general population when accessing services (PS, n = 32 [4, 19, 22–26, 28, 31, 41, 43, 44, 47, 51, 59, 60, 63, 65, 70–72, 78, 79, 81, 92, 97, 98, 101, 106, 107, 109, 112]; SR, n = 15 [122, 124, 127, 131, 135, 136, 138, 140, 142, 144, 148, 150–152]). The relevant data in these studies comprised of qualitative and non-comparative survey data.

**Table 13:** Rare diseases in UK primary studies and SRs relating to lack of knowledge and access to services

UK primary studies (n)	Systematic reviews (n)
Adrenal insufficiency (1) [97]	Childhood dementias (1), [138]
Ataxia and progressive ataxia (2) [43, 106]	CF (1) [148]
Desmoid fibromatosis (1) [60]	DMD (1) [124]
DMD (1) [41]	Fragile X syndrome (1) [131]
Dystonia (1) [72]	Inherited bleeding disorders, specifically haemophilia (1) [148]
Ehlers-Danlos syndrome (2) [25, 26]	Juvenile Idiopathic arthritis (1) [148]
Epidermolysis bullosa (1) [44]	MND (4) [122, 127, 140, 142]
Guillain–Barre syndrome (1) [19]	Nephrotic syndrome (1) [148]
Haemophilia (1) [109]	SCD (3) [123, 135, 144, 148]
Huntington disease (2) [24, 98]	Undiagnosed condition (1) [148]
IgA nephropathy (1) [107]	Multiple types of rare diseases (4: McMullan 2022; Tsitsani 2023; von der Lippe 2017; von der Lippe 2022) [136, 150–152]
Inherited bleeding disorders (2) [63, 65]	–
Lipoprotein lipase deficiency (1) [79]	–
Long-segment congenital tracheal stenosis (1) [112]	–
MND (2) [59, 81]	–
MSA (1) [78]	–
Phenylketonuria (1) [31]	–
Rare intellectual disabilities (1) [51]	–
SCD (5) [22, 23, 28, 71, 92]	–
Multiple rare diseases (4: Franklish 2022; McMullan 2022; Specialised Healthcare Alliance 2023; Spencer-Tansley 2018) [4, 47, 70, 101]	–

In addition to the perception that clinicians have a general lack of knowledge about rare diseases when accessing services, [22–25, 28, 31, 41, 43, 51, 59, 60, 63, 65, 70, 79, 81, 92, 97, 98, 101, 106, 112] qualitative data in primary studies indicated the perception that clinicians dismissed symptoms that they did not understand [26, 44, 107], and that they accused people with symptoms of a rare disease as exaggerating the extent of their symptoms, or entirely fabricating their symptoms, which was sometimes perceived to occur due to ignorance about rare diseases [71, 72, 79, 107]. As a result, PwRD and carers felt that they were not listened to or taken seriously [19, 47, 71, 72]. Data in SRs indicated similar experiences, with the perception that clinicians have a general lack of knowledge about rare diseases when accessing services [122, 131, 138, 140, 144, 150, 151]. Two SRs identified that clinicians’ lack of understanding around the specific need of MND and end of life meant that PwRD and carers were unable to have conversations about end of life [124, 127]. As a result, patients and their carers reported having to often educate healthcare professionals to receive the correct care [122, 131, 135, 136, 142].

#### Lack of information

Data on lack of information indicated that PwRD and their carers may receive less information about rare diseases listed in Table 14 when accessing services than the general population (PS, n = 22 [19, 21, 24, 26–28, 32, 37, 45, 51, 60, 75, 78, 79, 83, 84, 88, 93, 101, 103, 107, 110]; SR, n = 12 [122, 124, 137–140, 142, 143, 145, 149, 151, 153]) The relevant data in the studies comprised of qualitative data and non-comparative survey data.

**Table 14:** Rare diseases in UK primary studies and SRs relating to lack of information and access to services

UK primary studies (n)	Systematic reviews (n)
CF (1) [37]	Childhood dementia (1) [138]
CF diabetes (1) [45]	CF (1) [137]
Desmoid fibromatosis (1) [60]	DMD (2) [124, 143]
DMD (1) [93]	Frontotemporal lobar degeneration (1) [139]
Ehlers–Danlos syndrome (1) [26]	MND (4) [122, 140, 142, 153]
Guillain–Barre Syndrome (1) [19]	Rare epilepsy-related disorders and intellectual disability (1) [149]
Huntington disease (1) [24]	SCD (1) [145]
IgA Nephropathy (1) [107]	Multiple rare diseases (1: von der Lippe 2017) [151]
Lipoprotein lipase deficiency (1) [79]	–
MND (5) [83, 84, 88, 103, 110]	–
MSA (1) [78]	–
Rare intellectual disabilities [51]	–
SCD (3) [27, 28, 32]	–
Undiagnosed genetic conditions (1) [21]	–
Multiple rare diseases (2: Muir 2016; Spencer -Tansley 2018) [75, 101]	–

In addition to a general unmet need for information about rare diseases from service providers [19, 24, 26, 51, 60, 79, 83, 93, 101, 107], data in primary studies indicated a need for more information about what services and treatments were available [32, 45, 75, 84, 88, 110], who to contact with questions about rare diseases [21], more information about shielding during the COVID pandemic [27, 28], and information about sexual relationships [103]. Data in two SRs indicated similar experiences, including a lack of information specifically for carers [139, 142]. Of these, one SR reported quantitative survey data showing that carers of people with frontotemporal lobar degeneration are less satisfied with the information they receive than carers of people with Alzheimer’s disease, and that the information needs of frontotemporal lobar degeneration carers is higher than that of carers for people with Azheimer’s disease [139].

#### Lack of care co-ordination

Data indicated that PwRD listed in Table 15 and their carers experienced a lack of care co-ordination when accessing services (PS, n = 19 [21, 23, 25, 31, 47, 48, 52, 56, 60, 67, 73, 75, 78, 91, 96, 101, 105, 108, 110]; SR, n = 10 [114, 117, 122, 136, 138, 140, 143, 150–152]). The relevant data in these studies comprised of qualitative data and non-comparative survey data.

**Table 15:** Rare diseases in UK primary studies and SRs relating to lack of care coordination and access to services

UK primary studies (n)	Systematic reviews (n)
Alstrom syndrome (1) [48]	Childhood dementias (1) [138]
Chronic intestinal pseudo-obstruction (1) [48]	DMD (1) [143]
Deletion on chromosome 4q (1) [48]	Ehlers–Danlos syndrome (1) [114]
Desmoid fibromatosis (1) [60]	MND (2) [122, 140]
Ehlers–Danlos syndrome (2) [25, 48]	Multiple rare diseases (5: Assalone 2024; McMullan 2022; Tsitsani 2023; von der Lippe 2017; von der Lippe 2022) [117, 136, 150–152]
Hereditary spastic paraparesis (1) [52]	–
MND (3) [56, 91, 110]	–
MSA (1) [78]	–
Phenylketonuria (1) [31]	–
Polyneuropathy Organomegaly Endocrinopathy Monoclonal gammopathy Skin changes (POEMS) syndrome (1) [105]	–
SCD (2) [23, 48]	–
Undiagnosed genetic conditions (1) [21]	–
Studies which included multiple rare diseases (7: Franklish 2022; Limb 2010; Morris 2022; Muir 2016; Simpson 2021; Spencer-Tansley 2018; Walton 2023) [47, 67, 73, 75, 96, 101, 108]	–

Of the primary studies, 15 reported data relating to care co-ordination within health care services, including with respect to primary, secondary and tertiary services [21, 23, 25, 31, 47, 48, 52, 56, 60, 67, 73, 75, 78, 91, 101, 108]; and four studies reported data relating to care co-ordination between health and social care settings [78, 97, 105, 110]. Of the SRs, seven reported data relating to care co-ordination within health care settings, including with respect to primary, secondary and tertiary services [114, 136, 138, 140, 143, 150, 152]; and three SRs reported data relating to care co-ordination between health and social care settings [117, 122, 151].

Data in primary studies indicated that PwRD required multidisciplinary care which was perceived as lacking in coordination [21, 23, 31, 56, 67, 73, 75, 91, 96]. This sometimes meant that PwRD or their carers were required to take on a co-ordinating role themselves, communicating information between different health care professionals, and arranging multiple appointments at suitable times [21, 47, 48, 75, 78]. Data in SRs reported similar experiences, with reference to “fragmented care” and health professionals working in “silos” [114, 152].

### Access to services

#### General service access

Data indicated that PwRD listed in Table 16 and their carers experienced barriers to accessing services which were generically described as health care, without providing more detail about specific service settings (PS, n = 15 [19, 25, 27, 42, 47, 51, 52, 68–70, 76, 78, 91, 105, 110]; SR, n = 10 [116, 123, 124, 127, 131, 133, 138, 140, 143, 150]). The relevant data comprised of qualitative data, and comparative and non-comparative survey data.

**Table 16:** Rare diseases in UK primary studies and SRs relating to access to services (general)

UK primary studies (n)	Systematic reviews (n)
Ataxia (1) [42]	Childhood dementia (1) [138]
Ehlers–Danlos syndrome (1) [25]	DMD (2) [124, 143]
Genetic diseases (1) [69]	Fragile X syndrome (1) [131]
Guillain–Barre syndrome (1) [19]	MND (3) [116, 127, 140]
Hereditary spastic paraparesis (1) [52]	SCD (2) [123, 133]
MND (2) [91, 110]	Multiple rare diseases (1: Tsitsani 2023) [150]
MSA(2) [76, 78]	–
Polyneuropathy Organomegaly Endocrinopathy Monoclonal gammopathy Skin changes (POEMS) syndrome (1) [105]	–
SCD (1) [27]	–
Rare genetic intellectual diseases (2) [51]	–
Tuberous sclerosis complex (1) [68]	–
Multiple rare diseases (2: Franklish 2022; McMullan 2022) [47, 70]	–

Data in primary studies indicated a general lack of service provision. This was perceived as due to budgetary constraints [42], and a perceived need to “fight” or “struggle” to access services [51, 70]. Where there was limited service provision, PwRD and carers reported that they sometimes seek care via charities [19] or private care [25], and sometimes prefer to manage pain at home to avoid using NHS services which do not recognise their needs [27]. Follow up care after diagnosis or discharge from secondary or tertiary services was also perceived to be lacking [47, 52, 68, 69, 105]. Data in SRs reported similar experiences. Specific issues were raised in relation to primary care services [133, 150], with comparative data from one SR highlighting how the absence of primary care for patients with SCD (particularly children), increased their risk of hospitalization and 30-day re-admission compared to patients with other chronic conditions [133]. Barriers to accessing palliative and end of life care were also specifically raised by people living with MND and their carers [116, 127], as well as a lack of non-pharmacological and behavioral therapies for those living with childhood dementias [138].

#### Mental health services

Data indicated that PwRD listed in Table 17 and their carers experienced barriers to accessing mental health services (PS, n = 24 [19, 26–28, 31, 32, 36, 40, 44, 47, 49, 60, 67, 70, 76–79, 83, 97, 101, 102, 104, 110]; SR, n = 9 [70, 122, 128, 134, 138, 148–151]) The relevant data in these studies comprised of qualitative data and comparative and non-comparative survey data.

**Table 17:** Rare diseases in UK primary studies and SRs relating to access to services (mental health)

UK primary studies (n)	Systematic reviews (n)
22q11 deletion syndrome (1) [36]	Childhood dementias (1) [138]
Adrenal Insufficiency (1) [97]	CF (1) [148]
Desmoid fibromatosis (1) [60]	Inherited bleeding disorders, specifically haemophilia (1) [148]
DMD (1) [104]	Juvenile idiopathic arthritis (2) [134, 148]
Ehlers–Danlos syndrome (1) [26]	MND (2) [122, 128]
Epidermolysis Bullosa (1) [44]	Muscular dystrophies (1) [134]
Guillain–Barre Syndrome (1) [19]	Nephrotic syndrome (1) [148]
Lipoprotein lipase deficiency (1) [79]	Rare epilepsy related disorders (1) [149]
Mayer-Rokitansky-Kuster-Hauser syndrome (1) [49]	Rare intellectual disabilities (1) [149]
MND (2) [83, 110]	Rare or undiagnosed condition (not specified) (1) [148]
MSA (3) [76–78]	SCD (1) [148]
Phenylketonuria (1) [31]	Spina bifida (1) [134]
SCD (3) [27, 28, 32]	Multiple rare diseases (3: McMullan 2022; Tsitsani 2023; von der Lippe 2017) [70, 150, 151]
Multiple rare diseases (5: Crowe 2019; Franklish 2022; Limb 2010; McMullan 2022; Spencer-Tansley 2018) [40, 47, 67, 70, 101]	–

Data in primary studies indicated a general lack of psychological support to help with the mental health impact of coping with a rare disease. In particular, data in qualitative studies indicated the perception that clinicians do not consider the mental health impact of living with a rare disease [26–28, 44, 47], including for carers [78, 110]; and the perception that, when clinicians try to organise counselling, the waiting times are very long (in one study a 7 month waiting list was reported) [19] or it is not available, or that equivalent counselling services are available for more common diseases such as cancer [83]. Survey data showed that, of PwRDs included in studies relating to mental health, a minority had access to mental health support [31, 32, 76, 78, 83, 104], or access to sufficient mental health support [67]. SRs reported similar experiences, including the perception that carers of PwRD do not receive sufficient mental health support [138, 149].

These experiences potentially lead to inequity between the rare disease community and the general population, albeit difficulty accessing mental health services is also reported in the general population [154]. However, quantitative data in one primary study [104] and several SRs [118, 122, 134, 138, 139, 141, 147] showed that levels of anxiety, depression and stress are higher in the rare disease community than in the general population, potentially indicating greater need for mental health services.

One primary study reporting quantitative survey data found that, in a sample of 588 people with rare diseases who had accessed mental health services, 7% had accessed it through a specialist clinic (41/588) compared with 48% who had been referred by their GP (280/588) and 21% by clinicians at their hospital (123/588) [102]. This difference in referrals to mental health services may lead to inequity within the rare disease community.

#### Dental services

Data indicated that PwRD and carers experienced barriers to accessing dental services. Specifically, quantitative survey data in two UK primary studies indicated that people with rare inherited bleeding disorders find it difficult to find a dentist due to dental surgeries not accepting patients with a bleeding disorder [29, 63]. Both studies reported data relating to dental services in primary health care settings [29, 63].

#### Emergency services

Data indicated that PwRD and carers experienced barriers to accessing appropriate care in emergency services (PS, n = 2 [32, 92]; SR, n = 1 [123]). All studies reported data relating to people with SCD. Specifically, qualitative and quantitative survey data in primary studies indicated that people with SCD perceived delays to receiving appropriate pain management treatment in emergency service settings [32, 92]. SR data indicated similar experiences of pain management, and that SCD patients waited 25% longer than the general population and 50% longer than those with long bone fractures after considering race and triage priority [123].

#### Specialist services

Data indicated that PwRD listed in Table 18 and their carers experienced barriers to accessing specialist services (PS, n = 9 [24, 47, 67, 73–75, 87, 106, 111]; SR, n = 2 [114, 131]). Data in these studies comprised of qualitative data and non-comparative survey data.

**Table 18:** Rare diseases in UK primary studies and SRs relating to access to services (specialists)

UK primary studies (n)	Systematic reviews (n)
Acromegaly (1) [87]	Ehlers–Danlos syndrome (1) [114]
Ataxia and progressive ataxia (2) [74, 106]	Fragile X syndrome (1) [131]
CF (1) [111]	–
Huntington disease (1) [24]	–
Multiple rare diseases (4: Franklish 2022; Limb 2010; Morris 2022; Muir 2016) [47, 67, 73, 75]	–

Qualitative data in primary studies indicated that there were PwRD who did not have access to specialist centres. Sometimes they were not aware of specialist centres for their condition [67, 75], and sometimes there were significant delays to referral to specialist centres [47]. These studies did not explicitly mention distance to travel due to place of residence as a reason why they did not have access (see *place of residence*, below). Similarly, quantitative survey data reported that a minority of PwRD had access to specialist services [73–75, 106]. Data in SRs also indicated limited availability of specialist services [114, 131].

Furthermore, data indicated that PwRD may experience differences in access to specialist services. Quantitative survey data in one primary study indicated that people with ataxia with access to specialist ataxia centres had increased contact with a variety of different specialists (not limited to neurologists) compared with people with ataxia who were unable to access specialist ataxia centres [74]. Several primary studies reported quantitative survey data showing that a minority of PwRD had access to specialist centres [73, 74, 106, 108]. Rare diseases reported in these studies included ataxia [74, 106] and multiple rare diseases. This variation in access to specialist centres may lead to inequity within the rare disease community [73, 108].

#### Social care services

Data indicated that PwRD listed in Table 19 and their carers experienced barriers to accessing social care (PS, n = 12 [19, 36, 40, 47, 51, 67, 68, 78, 84, 85, 96, 98]; SR, n = 5 [121, 122, 128, 139, 151]). The relevant data in these studies comprised of qualitative data and non-comparative survey data.

**Table 19:** Rare diseases in UK primary studies and SRs relating to access to services (social care)

UK primary studies (n)	Systematic reviews (n)
22q11 deletion syndrome (1) [36]	Frontotemporal lobar degeneration (1) [139]
Guillain–Barre Syndrome (1) [19]	Huntington disease (1) [121]
Huntington disease (1) [98]	MND (2) [122, 129]
MND (2) [84, 85]	Multiple rare diseases (1: von der Lippe 2017) [151]
MSA (1) [78]	–
Rare intellectual disabilities (1) [51]	–
Tuberous sclerosis complex (1) [68]	–
Multiple rare diseases (4: Crowe 2019; Franklish 2022; Limb 2010; Simpson 2021) [40, 47, 67, 96]	–

Qualitative and quantitative survey data in primary studies indicated that PwRD wait a long time for access to social care services [36, 40, 68], and are sometimes refused services after long periods of needs assessment [47]. It was also indicated that social care workers are not familiar with rare diseases, and that there is sometimes a high turnover of staff, which can impact on the quality of care [51, 84, 85, 98]. SR data indicated similar experiences, including refusal of services [139, 151], lack of awareness of rare diseases amongst social care workers [121] and need for specific services for young people [122].

#### Services for undiagnosed conditions

Data on access to services for people with undiagnosed rare diseases indicated that people with symptoms of a rare disease but no diagnosis experienced barriers to accessing services. Two primary studies, including one qualitative study and one mixed methods study with quantitative survey data, reported data on undiagnosed conditions in this context [21, 75]. Data indicated that people with rare undiagnosed conditions perceived that services were unavailable or they did not know what services were available [21, 75]. This can lead to inequity between the rare disease community and general population, and within the rare disease community.

### Inequities experienced by specific groups in the rare disease community with respect to access to services

**Fig. 6:**
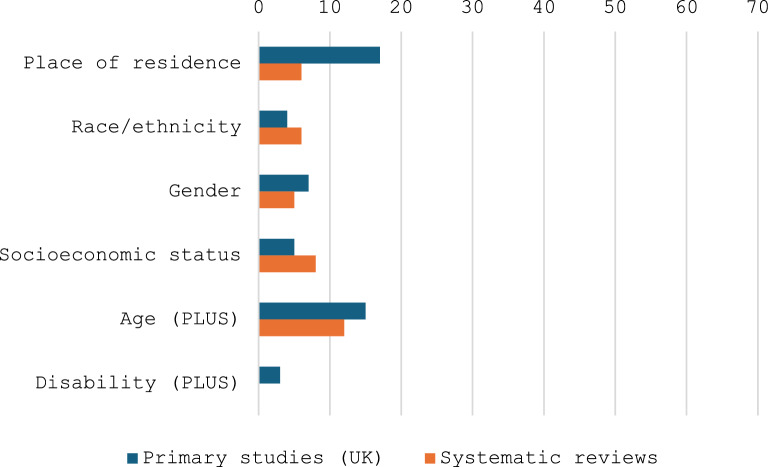
PROGRESS + inequities with respect to access to services

An overview of the numbers of studies indicating inequities in specific groups within the rare disease community with respect to access to services is presented in Fig. 6 [18].

#### Place of residence

Data on place of residence indicated that geographic location may impact on the experience of accessing services for PwRD listed in Table 20 or their carers (PS, n = 17 [4, 21, 35, 50, 52, 67, 74, 75, 78, 83, 84, 93, 94, 97, 98, 101, 106]; SR, n = 6 [119, 120, 132, 133, 145, 147]).

**Table 20:** Rare diseases in UK primary studies and SRs relating to place of residence and access to services

UK primary studies (n)	Systematic review (n)
Adrenal Insufficiency (1) [97]	Bleeding disorders (1) [147]
Ataxia and progressive ataxias (2) [74, 106]	SCD (4) [119, 132, 133, 145]
Congenital adrenal hyperplasia (1) [50]	Multiple non-cancer related rare diseases (1; Best 2022) [120]
DMD (1) [93]	–
Hereditary spastic paraparesis (1) [52]	–
Huntington’s disease (1) [98]	–
MND, including specifically ALS (2) [83, 85]	–
MSA (1) [78]	–
Osteogenesis imperfecta (1) [35]	–
Rare neurodegenerative conditions (1) [94]	–
Undiagnosed genetic conditions (1) [21]	–
Multiple rare diseases, including rare neurodegenerative conditions (5: Franklish 2022; Limb 2010; Muir 2016; Specialised Healthcare Alliance 2023; Spencer-Tansley 2018) [4, 47, 67, 75, 101]	–

Qualitative data in primary studies showed that PwRD and their carers need to travel long distances to access specialist services [4, 21, 52, 85, 97, 101, 120]. This was identified as particularly challenging for people in rural areas, as the specialist centres were typically located in large urban areas [52]. Data also suggested that services varied between locations, leading to different standards of care depending on place of residence [67, 83, 94]. SR evidence identified additional challenges for those living in rural areas who relied on public transport to access services [120].

Additionally, quantitative survey data identified in UK primary studies showed that PwRD and their carers who had to travel long distances to access services were less likely to attend clinics than those living nearer [35, 93], and were more likely to drop out of treatment plans [50]. This was shown to adversely affect other equity related experiences, for example, people who were unable to attend clinics due to distance were also less satisfied with the information they had access to [93]. Data also indicated that PwRD in the UK may have less access to services than people living in other European countries [74, 106]. One study reporting quantitative survey data found that people with Huntington’s disease in the UK were more likely to report dissatisfaction with clinicians’ knowledge than PwRD in the USA (UK = 82·6%; US = 64·3%) [98].

Quantitative data identified in SRs also reported that people living further away from specialist centres were less likely to attend than people living nearer [119, 132, 133].

#### Race/ethnicity

Data on ethnicity indicated that the ethnicity of PwRD listed in Table 21 may impact on their experience of accessing services (PS, n = 4 [27, 28, 92, 94]; SR, n = 6 [123, 133, 135, 141, 145, 146]).

**Table 21:** Rare diseases in UK primary studies and SRs relating to race/ethnicity and access to services

UK primary studies (n)	Systematic reviews (n)
SCD (3) [27, 28, 92]	SCD (6) [123, 133, 135, 141, 145, 146]
Multiple rare neurodegenerative conditions (1) [94]	–

Qualitative data in primary studies indicated that SCD patients thought there was a link between their ethnicity and health professionals’ dismissive attitudes towards how much pain they experienced [28, 92]. Furthermore, one study reported that, during the COVID19 pandemic, SCD patients felt that they were sometimes not treated as vulnerable due to their ethnicity [27]. SRs also indicated that people living with SCD felt stigmatized when accessing health care services, and the perception that stigmatization can impact the amount of funding which is allocated to research on SCD disease [135, 145, 146]. One SR reported the perception that people living with SCD experience delays to accessing health care due to their ethnicity [133].

Amongst people with rare neurodegenerative disorders, not speaking fluent English was identified as a barrier to accessing services [94].

#### Gender

Data indicated that the gender of PwRD listed in Table 22 or carers may impact on the experience of accessing services (PS, n = 7 [25, 30, 49, 64, 66, 103, 109]; SR, n = 5 [114, 130, 137, 144, 147]).

**Table 22:** Rare diseases in UK primary studies and SRs relating to gender and access to services

UK primary studies (n)	Systematic reviews (n)
CF (1) [30]	CF (2) [130, 137]
Ehlers–Danlos syndrome (1) [25]	Ehlers–Danlos syndrome (1) [114]
Inherited bleeding disorders (3) [64, 66, 109]	Inherited bleeding disorders (1) [147]
Mayer-Rokitansky-Kuster-Hauser syndrome (1) [49]	SCD (1) [144]
MND (1) [103]	–

Qualitative data in primary studies indicated that women with a rare disease sometimes felt dismissed by clinicians [25, 49, 66]. This included the perception that their concerns about symptoms were dismissed as related to mental illness [25], and the perception that there is a power imbalance between the patient and doctor which leads to feeling “pushed away” from accessing appropriate health care [49]. Qualitative UK primary studies also reported that women with a rare disease perceive a lack of support for issues relating to sexual and reproductive health, including that sexual and reproductive health is rarely discussed by health care professionals, and a lack of knowledge amongst clinicians on managing pregnancy [30, 103]. Similarly, qualitative data in SRs indicated that women with a rare disease perceive that women’s health is trivialised by some doctors [147]. It was also indicated that women’s health can be discomforting to discuss for both patients and clinicians, which leads to lack of open communication [137], and there was a perceived lack of support for issues relating to sexual and reproductive health. [114, 130, 144]

Additionally, quantitative survey data in one primary study reported that one third of women patients with menorrhagia, who also have a rare bleeding disorder, do not feel that they are supplied with sufficient information [64]. Quantitative survey data also reported that there was large divide between the level of knowledge in specialist clinicians and primary care clinicians, which can lead to substandard care for women not registered with specialist centres [109].

#### Socioeconomic status

Data indicated that the financial status of PwRD listed in Table 23 or carers may impact on the experience of accessing services (PS, n = 5 [25, 47, 65, 73, 98]; SR, n = 8 [114, 120, 122, 132, 133, 138, 145, 153]).

**Table 23:** Rare diseases in UK primary studies and SRs relating to socioeconomic status and access to services

UK primary studies (n)	Systematic reviews (n)
Ehlers–Danlos syndrome (1) [25]	ALS (1) [153]
Huntington disease (1) [98]	Childhood dementias (1) [138]
Inherited bleeding disorders (1) [65]	Ehlers–Danlos syndrome (1) [114]
Multiple rare diseases (2: Morris 2022, Franklish 2022) [47, 73]	MND (2) [122, 153]
**–**	SCD (3) [132, 133, 145]
**–**	Multiple non-cancer related rare diseases (1) [120]

Of the primary studies, all reported data relating to accessing private health or social care [25, 65, 73, 98]. Of the SRs, these included data relating to accessing health and social care in insurance-based health care systems outside the UK [114, 120, 122, 132, 133, 138, 145, 153].

Qualitative data in primary studies indicated that PwRD perceived unfairness with respect to access to services due to the need to pay for private services to avoid delays to care, or to receive a better standard of care [25, 73]. Qualitative data in SRs indicated barriers to accessing health care services for PwRDs and carers in countries with insurance-based health care systems [120, 145], and financial inequities arising from costs for non-medical needs such as childcare and time out of work when accessing services for rare diseases [132, 138, 153]. One SR reported that PwRD in the UK use private care at great cost although do not necessarily find it helpful [114].

Additionally, quantitative survey data in UK primary studies reported that PwRD and their carers (specifically, parents) find it difficult to pay for medical care and specialist equipment [65, 98]. One SR found that PwRDs on low incomes in insurance-based health care systems pay higher healthcare costs and use emergency services more frequently due to lack of appropriate insurance [133]. One SR reported survey data showing that the cost of informal care for spinal muscular atrophy increased with the severity of disease [122].

#### Age

Data indicated that the age of PwRD listed in Table 24 may impact on the experience of accessing services (PS, n = 15 [25, 27, 41, 48, 55, 62, 64, 67, 71, 75, 92, 93, 97, 99, 101]; SR, n = 12 [123–126, 131, 132, 138, 141, 145, 148–150]).

**Table 24:** Rare diseases in UK primary studies and SRs relating to age and access to services

UK primary studies (n)	Systematic reviews (n)
Adrenal Insufficiency (1) [97]	Childhood dementias (1) [138]
Alstrom syndrome (1) [48]	CF (3) [125, 126, 148]
Cavernoma (1) [48]	DMD (1) [124]
Chronic intestinal pseudo-obstruction (1) [48]	Fragile X syndrome (1) [131]
CF (1) [62]	Inherited bleeding disorders, specifically haemophilia (1) [148]
Deletion on chromosome 4q (1) [48]	Juvenile idiopathic arthritis (1) [148]
DMD (2) [41, 93]	Nephrotic syndrome (1) [148]
Ehlers–Danlos syndrome (2) [25, 48]	Rare epilepsy-related disorders and intellectual disability (1) [149]
Menorrhagia in the context of inherited bleeding disorders (1) [64]	Rare or undiagnosed condition (not specified) (1) [148]
Oesophageal atresia/tracheo-oesophageal fistula (1) [55]	SCD (5) [123, 132, 141, 145, 148]
SCD (4) [27, 48, 71, 92]	Multiple rare diseases (1: Tsitsani 2023) [150]
Tuberous sclerosis complex (1) [99]	–
Multiple rare diseases (3: Limb 2010; Muir 2016;Spencer-Tansley 2018) [67, 75, 101]	–

Qualitative data in primary studies reported the perception that children were treated differently to adults due to their age [25, 64, 92, 101]. This included the perception that young people were not given appropriate pain relief due to clinicians disregarding their claims about pain [92], and that young people were not given sufficient information about their care needs or treatment [64, 101]. It was also perceived that children cannot access appropriate support because some specialists are only accessible in adult services [25]. However, with respect to transitioning between paediatric and adult care, children’s services were perceived as better than adult services, and that the transition from child to adult services is not satisfactory [41, 48, 55, 62, 67, 75, 97]. It was also perceived that adults aged between 30 and 40 may have specific age-related needs similar to the specific needs of children or older adults but which are not catered for [27, 41]. SRs which reported qualitative data similarly reported the perception that children’s services were better than adult services, with a similar focus on the transition from children’s to adult’s services, where this difference was felt most acutely [124, 125, 131, 132, 138, 141, 148]. However, children and young adults also reported that their claims were sometimes dismissed or ignored by clinicians with respect to how they were treated [123, 126, 145].

Additionally, quantitative survey data in primary studies showed that adults are sometimes dissatisfied with adult rare disease services compared with children’s rare disease services [67, 75, 93, 99]. However, one survey, which was carried out at two time points, showed that the number of adults who were dissatisfied with adult rare disease services compared with children’s rare disease services had decreased from 30 to 16% between 2010 and 2016 [67, 75].

#### Disability

Data indicated that PwRD who also have disabilities may have different experiences of accessing services to able-bodied people (PS, n = 3) [51, 78, 81]. Rare diseases reported in these studies included MND [81], MSA [78] and rare genetic intellectual disabilities [51]. Data indicated that that carers perceive that clinicians lack understanding of people with a rare intellectual disability, which impacts on accessing services [51]. Data also indicated that carers of people with MND who are unable to meet their own basic needs due to disability were dissatisfied with the level of care whilst staying in hospital [81]. Also, mobility problems experienced by people with MSA were identified as leading to challenges when accessing services [78].

## Discussion

This scoping review summarises evidence relating to inequities experienced by the rare disease community with respect to receipt of a diagnosis and access to health and social care services. In particular, the review focuses on evidence from UK settings, but also includes international evidence from SRs. Overall, the review shows that the rare disease community’s experiences of receipt of a diagnosis and access to health and social care services are indicative of inequities compared with the general population, and within the rare disease community itself. In this section, we summarise some of the key findings, and the strengths and limitations, of the review.

### Inequities shared across the rare disease community

The majority of evidence was shared across the rare disease community, and indicative of inequity between the rare disease community and the general population. This included experiences of delayed diagnosis, often related to lack of knowledge of rare diseases amongst clinicians, experiences of lack of information provision, and experiences of lack of care coordination for patients with complex needs. Experiences of a lack of appropriate services across a range of different services were also, collectively, frequently reported. These experiences were shared amongst people with different types of rare diseases (based on UK data and often corroborated in the international literature in SRs), albeit some rare diseases occurred more frequently in the studies than others. In particular, all of the inequities associated with diagnosis except mental health services were supported with data from UK studies on MND; and studies on both MND and SCD were similarly prevalent amongst data on access to services. This may be partly due to them attracting more UK-based funding for research than less common rare diseases [14].

Although much of the data which were used to identify potential inequities between the rare disease community and general population were not comparative, it was sometimes apparent that the experiences were likely to be unique or relatively specific to the rare disease community. For example, the experience of lack of knowledge of rare diseases amongst clinicians is unlikely to be experienced by the general population with respect to more common diseases [155–157]. Relatedly, the experiences of delayed diagnosis may be exacerbated for the rare disease community compared with the general population due to lack of knowledge amongst clinicians [158]. (There is also a possibility that length of time to diagnosis stems from the complexity of clinical pathways required for diagnosis for some rare diseases, and thus may be attributable to recommended practice) [159, 160].

There was also evidence of experiences of poor-quality care amongst the rare disease community which may be experienced by others in the general population. Lack of care co-ordination, for example, may be experienced by others in the general population with complex needs [161]. Of the several types of services which were identified in the research as providing limited access, mental health services in the UK in particular is one where people in the general population also report limited access [154]. We included evidence relating to these experiences as indicating inequities in order to be inclusive of all the potentially relevant data. However, these findings should be interpreted more cautiously within the wider context of service access for the general population.

Whilst it may be reasonable to assume based on non-comparative data that the characteristics of rare diseases lead to inequities between the rare disease community and general population, it is harder to ascertain how these inequities are experienced within the rare disease community without comparative data, which was lacking in the literature. This included relatively few studies which compared lack of knowledge amongst clinicians, or access to information or services, across different types of rare disease. More research in this area is needed.

### Inequities experienced by specific groups in the rare disease community

The inequity data relating to specific groups within the rare disease community identified with reference to PROGRESS + revealed similar experiences to inequities shared across the rare disease community [18]. This included experiences of lack of information and lack of knowledge of clinicians in connection with rare diseases which affect children (e.g. childhood dementia), women (e.g. the impact of rare diseases on reproductive and menstrual health) and ethnic minorities (e.g. SCD). Furthermore, experiences of limited access to services for PwRDs were reported in connection with place of residence, socioeconomic status and age. However, for these groups, the experience of inequity may be exacerbated due to wider social determinants of health associated with the characteristics specified in PROGRESS + [18]. For example, the perception amongst women that the symptoms of rare diseases are dismissed by clinicians as part of normal menstrual health [66], and the perception amongst ethnic minorities that they are mistrusted by clinicians when experiencing pain related to SCD [28], both relate to a potential lack of knowledge amongst clinicians of the symptoms of rare diseases; additionally, with reference to PROGRESS +, they also indicate potentially unequal treatment of these groups within the rare disease community, which may further exacerbate the experience of inequity.

Structural and organisational factors relating to the provision of rare disease services across different geographical regions, and differences between adult and children services, also stratified the experience of receipt of a diagnosis and access to services within the rare disease community. Children’s services were typically perceived as better than adult services. This does not, however, mean there are not challenges with children’s services. Collecting data from children can be challenging, and this may lead to children being a silent majority in the assessment of rare disease services [162].

## Implications for research and practice

### Research

Whilst we identified many studies which reported poor-quality experiences amongst PwRD with respect to diagnosis and access to services, these studies were not typically explicitly aiming to identify issues relating to inequity. Thus, the experiences of inequity we identified were reported almost incidentally amongst wider discussion of the experiences of living or caring for someone with a rare disease. More comparative research is needed which compares the experiences of diagnosis and access to services between the general population and rare disease community, and within the rare disease community for different types of rare disease, including with reference to wider social determinants of health set out in PROGRESS +  [18].

There is also a particular need for more research on inequities relating to accessing social care services, which was relatively underrepresented compared with accessing health care services. We also identified relatively few studies on the prevalence of ethnic minority data in genetic data sets, which we have been alerted to by topic experts as a major concern for equity relating to receipt of a diagnosis (personal correspondence). We are aware of some studies in this area using genetic datasets in the USA, and there may be a need for more research in this area in the UK [163, 164]. A recent report by the UK NHS Race and Health Observatory does consider ethnic inequities in genomics, but the disease focus is broader than our review, including cancer, inherited and common conditions, in addition to rare conditions [165].

### Practice

The evidence gathered in this review suggests that rare diseases should continue to be considered for inclusion in the Core20PLUS5 framework to increase action to reduce health inequalities. In a health care system that is designed for high volume patient groups and severely resource constrained, PwRD are particularly vulnerable [166]. We are aware of work underway which is addressing some of the identified inequities, which future work can build on. Lack of knowledge amongst clinicians can be addressed through education and readily accessible information sources for clinicians on rare diseases. This is being developed by the National Genomics Education Programme, including the GeNotes tool which provides clinicians with information on rare genetic diseases [167], and the Rare Disease Education Hub, which is developing targeted educational interventions for clinicians, including for both genomic and non-genomic forms of rare disease [168]. Artificial intelligence may also help to match symptoms with potential diagnoses [169]. Lack of information for patients can be addressed through partnerships between patient groups and clinicians to develop relevant and accessible information sources. This is ongoing through organisations such as Unique (https://rarechromo.org/), who develop patient information for rare chromosome and genetic disorders which is peer reviewed by clinicians.

Lack of care co-ordination can be addressed through the rare disease collaborative networks (RDCNs). RDCNs consist of groups of providers with a shared interest in developing understanding of a particular rare disease or set of rare diseases, and work together to further research, increase knowledge and improve the patient experience for PwRDs [2]. Clinical networks such as these will be embedded in NHS service specifications, and underpinned by commissioned research on how best to operationalise better co-ordination of care in the NHS. Mental health service access is starting to be addressed through the requirement for new and revised NHS service specifications to consider the psychosocial needs of patients, including for PwRD. Furthermore, the aforementioned GeNotes tool will soon include education resources on mental health and psychological services to support those living with rare conditions, their families, and carers [2].

Age-related concerns relating to transition from paediatric to adult care are being addressed by the Children and Young People’s Transformation Programme, which is developing a framework to aid the design of transition pathways that improve health outcomes [2]. Service access for people with undiagnosed conditions is starting to be addressed through a pilot test of two SWAN (Syndrome Without A Name) clinics in England, one for children and one for adults, drawing on experience of SWAN clinics in Wales [2]. With fewer patients to consider, SWAN clinics be relatively less resource intensive than high volume clinics and pilot tests may be more scalable.

### Strengths and limitations

To the best of our knowledge, the current review is more extensive in its coverage of different types of rare diseases and the inequities experienced than any other scoping or SRs. We identified 40 SRs which explored inequities relating to rare diseases, but none were as extensive in their coverage of rare diseases or types of inequity. It is a limitation that the primary studies were limited to UK studies, which reflected the interest of the commissioner for whom the review was carried out. The data were, however, supplemented with the international literature via SRs. The 2010 date limit is a limitation, but has likely made the findings more relevant to the contemporary context. The inclusion of data which is not explicitly comparative about areas of potential inequity is a limitation, which means we have framed some of the data more cautiously as indicative of inequity.

## Conclusion

This review has drawn attention to experiences of the rare disease community with respect to receipt of a diagnosis and access to services which are different to experiences in the general population, and within the rare disease community itself. Some of these experiences are clearly attributable to factors which are unfair, avoidable and systemic, in particular those which are experienced by specific groups within the rare disease community identified with reference to PROGRESS + [18]. Experiences which are shared across the rare disease community, relating to delayed diagnosis, lack of knowledge, information, care co-ordination and access to various services, also appeared to indicate inequity. Overall, the general population are unlikely to have similar experiences of diagnosis or service access, as these problems are less likely to be encountered for more common diseases.

## Supplementary Information

Additional file 1.

Additional file 2.

Additional file 3.

## Data Availability

The datasets used and analysed during the current study are available from the corresponding author on reasonable request.

## Abbreviations

ALS: Amyotrophic lateral sclerosis
DHSC: Department of health and social care
DMD: Duchenne muscular dystrophy
EU: European Union
HCP: Healthcare practiioner
ICS: Integrated care systems
IPF: Idiopathic pulmonary fibrosis
MeSH: Medical subject headings
MND: Motor neuron disease
MRKH: Mayer-Rokitansky-Kuster-Hauser syndrome
MSA: Multiple system atrophy
NHS: National Health Service
NIHR: National Institute for Health and Social Care Research
PICo: Population/problem, phenomenon of interest, context
PERSPEX: Public engagement in research for health and social policy at Exeter
POEMS: Polyneuropathy organomegaly endocrinopathy monoclonal gammopathy skin changes
PRISMA: Preferred reporting items for systematic reviews and meta-analyses
PROGRESS +: Place of residence, race/ethnicity, gender, religion, education, socioeconomic status, social capital (+ age, disability)
PRP: Policy research programme
PS: Primary study
PwRD: People with a rare disease
RDCN: Rare disease collaborative network
SCD: Sickle cell disease
SR: Systematic review
UK: United Kingdom
USA: Unite States of America
