# Supplementary material for: Evidence of inequities experienced by the rare disease community with respect to receipt of a diagnosis and access to services: a scoping review of UK and international evidence
Source: Orphanet J Rare Dis. 2025 Jun 12;20:303. doi: 10.1186/s13023-025-03818-w (PMC12164139; doi:10.1186/s13023-025-03818-w)
Supplement: Supplementary file 1 — Additional file 1. [file 13023_2025_3818_MOESM1_ESM.docx]

Supplementary File 1.

Bibliographic database search strategies

Database: ASSIA
Host: ProQuest
Issue: n/a
Date Searched: 7^th^ February 2024
Searcher: SB
Hits: 478
Strategy:

1. MAINSUBJECT.EXACT("Rare diseases") OR ABSTRACT,TITLE((rare near/1 (autoimmune or autosomal or blood or bone or cardia* or cardio* or childhood or chromosom* or CNV or condition* or congenital or connective or contag* or "copy number variant" or derma* or develop* or disease* or disorder* or dominant or familial or frequency or gene* or genotype or haplotype or hereditary or immun* or inflammatory or inherited or kidney or liver or mendelian or metabolic or metasta* or monogenic or muscul* or mutat* or neuro* or paediatric or pathogen or pediatric or phenotype or polygenic or recessive or respira* or skeletal or skin or tumo* or uro* or variant* or "x linked" or zoono*)) OR "highly specialised technolog*" OR (orphan near/1 (disease* or drug* or medicine* or medicinal)) OR orphanet OR "syndrome* without a name" OR (("low frequency" or neglected) near/2 (condition* or disease or disorder*)) OR (ultraorphan or ultrarare))
2. MAINSUBJECT.EXACT("Genetic disorders") OR ABSTRACT,TITLE((gene or genes or genetic* or genomic*) near/1 (condition* or disease* or disorder* or medicine or rare))
3. MAINSUBJECT.EXACT("Health care access") OR MAINSUBJECT.EXACT("Misdiagnosis") OR ABSTRACT,TITLE((access* or availab* or entry or referral* or pathway* or uptake or utili?ation) near/3 (care or delay* or diagnos* or healthcare or secondary or service* or specialist or support* or time)) OR (diagnos* near/3 (delay* or error* or incorrect* or missed or time or specialist* or support)) OR misdiagnos* OR ((healthcare or "health care" or "health service*" or information* or specialist*) near/2 (need* or support*)) OR ((GP* or "general practitioner*" or “primary care”) near/1 referral*)
4. MAINSUBJECT.EXACT("Health disparities") OR MAINSUBJECT.EXACT("Socioeconomic status") OR ABSTRACT,TITLE((inequalit* or inequit* or equalit* or equit* or divers* or discriminat* or disadvantage* or barrier* or obstacle* or utili*ation or unjust* or unfair* or underserved or minorit* or stigma* or disparit* or ethnic* or race or racial* or racis* or SES or SEP or sociodemographic* or "socio-demographic*" or income or wealth* or literacy or poverty or education or "educational level" or "educational attainment" or "well educated" or "better educated" or unemploy* or "home owner*" or tenure or affluen* or "well off" or "better off" or "worse off" or communication or information or informed or knowledge or geograph* or regional or postcode or rural) or (health near/2 (gap* or gradient* or hierarch*)) or (unmet near/2 need*) or (financial near/2 (resources or situation)))
5. 1 OR 2
6. 3 AND 4 AND 5

Database: CINAHL
Host: EBSCO
Issue: n/a
Date Searched: 7^th^ February 2024
Searcher: SB
Hits: 6673
Strategy:

1. TI ( (rare N1 (autoimmune or autosomal or blood or bone or cardia* or cardio* or childhood or chromosom* or CNV or condition* or congenital or connective or contag* or "copy number variant" or derma* or develop* or disease* or disorder* or dominant or familial or frequency or gene* or genotype or haplotype or hereditary or immun* or inflammatory or inherited or kidney or liver or mendelian or metabolic or metasta* or monogenic or muscul* or mutat* or neuro* or paediatric or pathogen or pediatric or phenotype or polygenic or recessive or respira* or skeletal or skin or tumo* or uro* or variant* or "x linked" or zoono*)) ) OR AB ( (rare N1 (autoimmune or autosomal or blood or bone or cardia* or cardio* or childhood or chromosom* or CNV or condition* or congenital or connective or contag* or "copy number variant" or derma* or develop* or disease* or disorder* or dominant or familial or frequency or gene* or genotype or haplotype or hereditary or immun* or inflammatory or inherited or kidney or liver or mendelian or metabolic or metasta* or monogenic or muscul* or mutat* or neuro* or paediatric or pathogen or pediatric or phenotype or polygenic or recessive or respira* or skeletal or skin or tumo* or uro* or variant* or "x linked" or zoono*)) )
2. TI "highly specialised technolog*" OR AB "highly specialised technolog*"
3. TI ( (orphan N1 (disease* or drug* or medicine* or medicinal)) ) OR AB ( (orphan N1 (disease* or drug* or medicine* or medicinal)) )
4. TI orphanet OR AB orphanet
5. TI "syndrome* without a name" OR AB "syndrome* without a name"
6. TI ( (("low frequency" or neglected) N2 (condition* or disease or disorder*)) ) OR AB ( (("low frequency" or neglected) N2 (condition* or disease or disorder*)) )
7. TI ( (ultraorphan or ultrarare) ) OR AB ( (ultraorphan or ultrarare) )
8. (MH "Rare Diseases")
9. (MH "Drugs, Orphan")
10. S1 OR S2 OR S3 OR S4 OR S5 OR S6 OR S7 OR S8 OR S9
11. TI ( ((gene or genes or genetic* or genomic*) N1 (condition* or disease* or disorder* or medicine or rare)) ) OR AB ( ((gene or genes or genetic* or genomic*) N1 (condition* or disease* or disorder* or medicine or rare)) )
12. (MH "Sequence Analysis+")
13. (MH "Hereditary Diseases+")
14. S11 OR S12 OR S13
15. TI ( (access* or availab* or entry or referral* or pathway* or uptake or utili#ation) N3 (care or delay* or diagnos* or healthcare or secondary or service* or specialist or support* or time) ) OR AB ( (access* or availab* or entry or referral* or pathway* or uptake or utili#ation) N3 (care or delay* or diagnos* or healthcare or secondary or service* or specialist or support* or time) )
16. TI ( diagnos* N3 (delay* or error* or incorrect* or missed or time or specialist* or support) ) OR AB ( diagnos* N3 (delay* or error* or incorrect* or missed or time or specialist* or support) )
17. TI misdiagnos* OR AB misdiagnos*
18. TI ( (healthcare or "health care" or "health service*" or information* or specialist*) N2 (need* or support*) ) OR AB ( (healthcare or "health care" or "health service*" or information* or specialist*) N2 (need* or support*) )
19. TI ( (GP* or "general practitioner*" or primary care) N1 referral* ) OR AB ( (GP* or "general practitioner*" or primary care) N1 referral* )
20. (MH "Health Care Delivery+")
21. (MH "Diagnosis, Delayed")
22. (MH "Diagnostic Errors")
23. (MH "Failure to Diagnose")
24. S15 OR S16 OR S17 OR S18 OR S19 OR S20 OR S21 OR S22 OR S23
25. S10 OR S14
26. S24 AND S25
27. TI ( (gene or genes or genetic* or genomic* or "exome sequencing") N3 (counsel* or diagnos* or service* or test*) ) OR AB ( (gene or genes or genetic* or genomic* or "exome sequencing") N3 (counsel* or diagnos* or service* or test*) )
28. (MH "Genetic Counseling")
29. (MH "Genetic Screening")
30. S27 OR S28 OR S29
31. TI ( access* or availab* or delay* or distribution or entry or need or needs or referral* or pathway* or receiving or uptake or utili#ation ) OR AB ( access* or availab* or delay* or distribution or entry or need or needs or referral* or pathway* or receiving or uptake or utili#ation )
32. (MH "Health Care Delivery+")
33. S31 OR S32
34. S30 AND S33
35. TI ( inequalit* or inequit* or equalit* or equit* or disparit* or divers* or discriminat* or disadvantage* or barrier* or obstacle* or utili#ation or unjust* or unfair* or underserved or minorit* or stigma* ) OR AB ( inequalit* or inequit* or equalit* or equit* or disparit* or divers* or discriminat* or disadvantage* or barrier* or obstacle* or utili#ation or unjust* or unfair* or underserved or minorit* or stigma* )
36. TI ( ethnic* or race or racial* or racis* ) OR AB ( ethnic* or race or racial* or racis* )
37. TI ( (social* or "socio-economic" or socioeconomic or economic or structural or material) N2 (advantage* or disadvantage* or exclude* or exclusion or include* or inclusion or status or position or gradient* or hierarch* or class* or determinant*) ) OR AB ( (social* or "socio-economic" or socioeconomic or economic or structural or material) N2 (advantage* or disadvantage* or exclude* or exclusion or include* or inclusion or status or position or gradient* or hierarch* or class* or determinant*) )
38. TI ( health N2 (gap* or gradient* or hierarch*) ) OR AB ( health N2 (gap* or gradient* or hierarch*) )
39. (MH "Healthcare Disparities")
40. (MH "Health Status Disparities+")
41. (MH "Health Inequities")
42. (MH "Urban Population")
43. (MH "Rural Population")
44. (MH "Stigma")
45. (MH "Socioeconomic Factors+") OR (MH "Communication")
46. TI ( SES or SEP or sociodemographic* or "socio-demographic*" or income or wealth* or literacy or poverty or education or "educational level" or "educational attainment" or "well educated" or "better educated" or unemploy* or "home owner*" or tenure or affluen* or "well off" or "better off" or "worse off" ) OR AB ( SES or SEP or sociodemographic* or "socio-demographic*" or income or wealth* or literacy or poverty or education or "educational level" or "educational attainment" or "well educated" or "better educated" or unemploy* or "home owner*" or tenure or affluen* or "well off" or "better off" or "worse off" )
47. TI unmet N2 need* OR AB unmet N2 need*
48. TI ( financial N0 (resources or situation) ) OR AB ( financial N0 (resources or situation) )
49. TI ( geograph* or regional or postcode or rural ) OR AB ( geograph* or regional or postcode or rural )
50. TI ( communication or information or informed or knowledge ) OR AB ( communication or information or informed or knowledge )
51. S35 OR S36 OR S37 OR S38 OR S39 OR S40 OR S41 OR S42 OR S43 OR S44 OR S45 OR S46 OR S47 OR S48 OR S49 OR S50
52. S26 OR S34
53. S51 AND S52

Notes: Limiters - Publication Date: 20100101-20241231

Database: Embase
Host: Ovid
Issue: 1974 to 2024 February 05
Date Searched: 6^th^ February 2024
Searcher: SB
Hits: 15631
Strategy:

1. (rare adj2 (autoimmune or autosomal or blood or bone or cardia* or cardio* or childhood or chromosom* or CNV or condition* or congenital or connective or contag* or "copy number variant" or derma* or develop* or disease* or disorder* or dominant or familial or frequency or gene* or genotype or haplotype or hereditary or immun* or inflammatory or inherited or kidney or liver or mendelian or metabolic or metasta* or monogenic or muscul* or mutat* or neuro* or paediatric or pathogen or pediatric or phenotype or polygenic or recessive or respira* or skeletal or skin or tumo* or uro* or variant* or "x linked" or zoono*)).tw,kw.
2. "highly specialised technolog*".tw,kw.
3. (orphan adj2 (disease* or drug* or medicine* or medicinal)).tw,kw.
4. orphanet.tw,kw.
5. "syndrome* without a name".tw,kw.
6. (("low frequency" or neglected) adj3 (condition* or disease or disorder*)).tw,kw.
7. (ultraorphan or ultrarare).tw,kw.
8. rare disease/
9. orphan drug/
10. or/1-9
11. ((gene or genes or genetic* or genomic*) adj2 (condition* or disease* or disorder* or medicine or rare)).tw,kw.
12. dna sequencing/
13. exp genetic disorder/
14. or/11-13
15. 10 or 14
16. ((access* or availab* or entry or referral* or pathway* or uptake or utili?ation) adj4 (care or delay* or diagnos* or healthcare or secondary or service* or specialist or support* or time)).tw.
17. (diagnos* adj4 (delay* or error* or incorrect* or missed or time or specialist* or support)).tw.
18. misdiagnos*.tw.
19. ((healthcare or "health care" or "health service*" or information* or specialist*) adj3 (need* or support*)).tw.
20. ((GP* or "general practitioner*" or primary care) adj2 referral*).tw.
21. exp health care access/
22. exp health care delivery/
23. delayed diagnosis/
24. or/16-23
25. 15 and 24
26. ((gene or genes or genetic* or genomic* or "exome sequencing") adj4 (counsel* or diagnos* or service* or test*)).tw,kw.
27. exp genetic service/
28. or/26-27
29. (access* or availab* or delay* or distribution or entry or need or needs or referral* or pathway* or receiving or uptake or utili?ation).tw.
30. exp health care access/
31. exp health care delivery/
32. or/29-31
33. 28 and 32
34. 25 or 33
35. (inequalit* or inequit* or equalit* or equit* or disparit* or divers* or discriminat* or disadvantage* or barrier* or obstacle* or utili*ation or unjust* or unfair* or underserved or minorit* or stigma*).tw.
36. (ethnic* or race or racial* or racis*).tw.
37. ((social* or "socio-economic" or socioeconomic or economic or structural or material) adj3 (advantage* or disadvantage* or exclude* or exclusion or include* or inclusion or status or position or gradient* or hierarch* or class* or determinant*)).tw.
38. (health adj3 (gap* or gradient* or hierarch*)).tw.
39. exp vulnerable population/
40. exp social stigma/
41. exp socioeconomics/
42. social class/
43. health care disparity/
44. health disparity/
45. urban population/
46. (SES or SEP or sociodemographic* or "socio-demographic*" or income or wealth* or literacy or poverty or education or "educational level" or "educational attainment" or "well educated" or "better educated" or unemploy* or "home owner*" or tenure or affluen* or "well off" or "better off" or "worse off").tw.
47. (unmet adj3 need*).tw.
48. (financial adj1 (resources or situation)).tw.
49. (geograph* or regional or postcode or rural).tw.
50. (communication or information or informed or knowledge).tw.
51. interpersonal communication/
52. or/35-51
53. exp United Kingdom/
54. (national health service* or nhs*).ti,ab,in,ad.
55. (english not ((published or publication* or translat* or written or language* or speak* or literature or citation*) adj5 english)).ti,ab.
56. (gb or "g.b." or britain* or (british* not "british columbia") or uk or "u.k." or united kingdom* or (england* not "new england") or northern ireland* or northern irish* or scotland* or scottish* or ((wales or "south wales") not "new south wales") or welsh*).ti,ab,jx,in,ad.
57. (bath or "bath's" or ((birmingham not alabama*) or ("birmingham's" not alabama*) or bradford or "bradford's" or brighton or "brighton's" or bristol or "bristol's" or carlisle* or "carlisle's" or (cambridge not (massachusetts* or boston* or harvard*)) or ("cambridge's" not (massachusetts* or boston* or harvard*)) or (canterbury not zealand*) or ("canterbury's" not zealand*) or chelmsford or "chelmsford's" or chester or "chester's" or chichester or "chichester's" or coventry or "coventry's" or derby or "derby's" or (durham not (carolina* or nc)) or ("durham's" not (carolina* or nc)) or ely or "ely's" or exeter or "exeter's" or gloucester or "gloucester's" or hereford or "hereford's" or hull or "hull's" or lancaster or "lancaster's" or leeds* or leicester or "leicester's" or (lincoln not nebraska*) or ("lincoln's" not nebraska*) or (liverpool not (new south wales* or nsw)) or ("liverpool's" not (new south wales* or nsw)) or ((london not (ontario* or ont or toronto*)) or ("london's" not (ontario* or ont or toronto*)) or manchester or "manchester's" or (newcastle not (new south wales* or nsw)) or ("newcastle's" not (new south wales* or nsw)) or norwich or "norwich's" or nottingham or "nottingham's" or oxford or "oxford's" or peterborough or "peterborough's" or plymouth or "plymouth's" or portsmouth or "portsmouth's" or preston or "preston's" or ripon or "ripon's" or salford or "salford's" or salisbury or "salisbury's" or sheffield or "sheffield's" or southampton or "southampton's" or st albans or stoke or "stoke's" or sunderland or "sunderland's" or truro or "truro's" or wakefield or "wakefield's" or wells or westminster or "westminster's" or winchester or "winchester's" or wolverhampton or "wolverhampton's" or (worcester not (massachusetts* or boston* or harvard*)) or ("worcester's" not (massachusetts* or boston* or harvard*)) or (york not ("new york*" or ny or ontario* or ont or toronto*)) or ("york's" not ("new york*" or ny or ontario* or ont or toronto*))))).ti,ab,in,ad.
58. (bangor or "bangor's" or cardiff or "cardiff's" or newport or "newport's" or st asaph or "st asaph's" or st davids or swansea or "swansea's").ti,ab,in,ad.
59. (aberdeen or "aberdeen's" or dundee or "dundee's" or edinburgh or "edinburgh's" or glasgow or "glasgow's" or inverness or (perth not australia*) or ("perth's" not australia*) or stirling or "stirling's").ti,ab,in,ad.
60. (armagh or "armagh's" or belfast or "belfast's" or lisburn or "lisburn's" or londonderry or "londonderry's" or derry or "derry's" or newry or "newry's").ti,ab,in,ad.
61. or/53-60
62. (exp "arctic and antarctic"/ or exp oceanic regions/ or exp western hemisphere/ or exp africa/ or exp asia/ or exp "australia and new zealand"/) not (exp United Kingdom/ or europe/)
63. 61 not 62
64. 34 and 52 and 63
65. ((systematic or Cochrane or effectiveness or qualitative or mapping or overview or realist or scoping or umbrella) adj2 review*).tw.
66. (metasynthes?s or "meta synthes?s" or "meta ethnography" or "meta analys?s").tw.
67. ((integrative or integrated) adj1 review*).tw.
68. ((evidence or research) adj1 synthes?s).tw.
69. or/65-68
70. 34 and 52 and 69
71. 63 or 69
72. limit 71 to yr="2010 -Current"

Database: HMIC
Host: Ovid
Issue: 1979 to November 2023
Date Searched: 6^th^ February 2024
Searcher: SB
Hits: 176
Strategy:

1. (rare adj2 (autoimmune or autosomal or blood or bone or cardia* or cardio* or childhood or chromosom* or CNV or condition* or congenital or connective or contag* or "copy number variant" or derma* or develop* or disease* or disorder* or dominant or familial or frequency or gene* or genotype or haplotype or hereditary or immun* or inflammatory or inherited or kidney or liver or mendelian or metabolic or metasta* or monogenic or muscul* or mutat* or neuro* or paediatric or pathogen or pediatric or phenotype or polygenic or recessive or respira* or skeletal or skin or tumo* or uro* or variant* or "x linked" or zoono*)).tw.
2. "highly specialised technolog*".tw.
3. (orphan adj2 (disease* or drug* or medicine* or medicinal)).tw.
4. orphanet.tw.
5. "syndrome* without a name".tw.
6. (("low frequency" or neglected) adj3 (condition* or disease or disorder*)).tw.
7. (ultraorphan or ultrarare).tw.
8. or/1-7
9. ((gene or genes or genetic* or genomic*) adj2 (condition* or disease* or disorder* or medicine or rare)).tw.
10. 8 or 9
11. ((access* or availab* or entry or referral* or pathway* or uptake or utili?ation) adj4 (care or delay* or diagnos* or healthcare or secondary or service* or specialist or support* or time)).tw.
12. (diagnos* adj4 (delay* or error* or incorrect* or missed or time or specialist* or support)).tw.
13. misdiagnos*.tw.
14. ((healthcare or "health care" or "health service*" or information* or specialist*) adj3 (need* or support*)).tw.
15. ((GP* or "general practitioner*" or primary care) adj2 referral*).tw.
16. or/11-15
17. 10 and 16
18. ((gene or genes or genetic* or genomic* or "exome sequencing") adj4 (counsel* or diagnos* or service* or test*)).tw.
19. (access* or availab* or delay* or distribution or entry or need or needs or referral* or pathway* or receiving or uptake or utili?ation).tw.
20. 18 and 19
21. 17 or 20
22. (inequalit* or inequit* or equalit* or equit* or disparit* or divers* or discriminat* or disadvantage* or barrier* or obstacle* or utili*ation or unjust* or unfair* or underserved or minorit* or stigma*).tw.
23. (ethnic* or race or racial* or racis*).tw.
24. ((social* or "socio-economic" or socioeconomic or economic or structural or material) adj3 (advantage* or disadvantage* or exclude* or exclusion or include* or inclusion or status or position or gradient* or hierarch* or class* or determinant*)).tw.
25. (health adj3 (gap* or gradient* or hierarch*)).tw.
26. (SES or SEP or sociodemographic* or "socio-demographic*" or income or wealth* or literacy or poverty or education or "educational level" or "educational attainment" or "well educated" or "better educated" or unemploy* or "home owner*" or tenure or affluen* or "well off" or "better off" or "worse off").tw.
27. (unmet adj3 need*).tw.
28. (financial adj1 (resources or situation)).tw.
29. (geograph* or regional or postcode or rural).tw.
30. (communication or information or informed or knowledge).tw.
31. or/22-30
32. 21 and 31

Database: MEDLINE
Host: Ovid
Issue: 1946 to February 05, 2024
Date Searched: 6^th^ February 2024
Searcher: SB
Hits: 3837
Strategy:

1. (rare adj2 (autoimmune or autosomal or blood or bone or cardia* or cardio* or childhood or chromosom* or CNV or condition* or congenital or connective or contag* or "copy number variant" or derma* or develop* or disease* or disorder* or dominant or familial or frequency or gene* or genotype or haplotype or hereditary or immun* or inflammatory or inherited or kidney or liver or mendelian or metabolic or metasta* or monogenic or muscul* or mutat* or neuro* or paediatric or pathogen or pediatric or phenotype or polygenic or recessive or respira* or skeletal or skin or tumo* or uro* or variant* or "x linked" or zoono*)).tw,kw.
2. "highly specialised technolog*".tw,kw.
3. (orphan adj2 (disease* or drug* or medicine* or medicinal)).tw,kw.
4. orphanet.tw,kw.
5. "syndrome* without a name".tw,kw.
6. (("low frequency" or neglected) adj3 (condition* or disease or disorder*)).tw,kw.
7. (ultraorphan or ultrarare).tw,kw.
8. Rare Diseases/
9. Orphan Drug Production/
10. or/1-9
11. ((gene or genes or genetic* or genomic*) adj2 (condition* or disease* or disorder* or medicine or rare)).tw,kw.
12. exp Sequence Analysis, DNA/
13. exp Genetic Diseases, Inborn/
14. or/11-13
15. 10 or 14
16. ((access* or availab* or entry or referral* or pathway* or uptake or utili?ation) adj4 (care or delay* or diagnos* or healthcare or secondary or service* or specialist or support* or time)).tw.
17. (diagnos* adj4 (delay* or error* or incorrect* or missed or time or specialist* or support)).tw.
18. misdiagnos*.tw.
19. ((healthcare or "health care" or "health service*" or information* or specialist*) adj3 (need* or support*)).tw.
20. ((GP* or "general practitioner*" or primary care) adj2 referral*).tw.
21. exp Health Services Accessibility/
22. exp "Delivery of Health Care"/
23. delayed diagnosis/
24. or/16-23
25. 15 and 24
26. ((gene or genes or genetic* or genomic* or "exome sequencing") adj4 (counsel* or diagnos* or service* or test*)).tw,kw.
27. exp Genetic Services/
28. or/26-27
29. (access* or availab* or delay* or distribution or entry or need or needs or referral* or pathway* or receiving or uptake or utili?ation).tw.
30. exp Health Services Accessibility/
31. exp "Delivery of Health Care"/
32. or/29-31
33. 28 and 32
34. 25 or 33
35. (inequalit* or inequit* or equalit* or equit* or disparit* or divers* or discriminat* or disadvantage* or barrier* or obstacle* or utili*ation or unjust* or unfair* or underserved or minorit* or stigma*).tw.
36. (ethnic* or race or racial* or racis*).tw.
37. ((social* or "socio-economic" or socioeconomic or economic or structural or material) adj3 (advantage* or disadvantage* or exclude* or exclusion or include* or inclusion or status or position or gradient* or hierarch* or class* or determinant*)).tw.
38. (health adj3 (gap* or gradient* or hierarch*)).tw.
39. Vulnerable populations/
40. social stigma/
41. socioeconomic factors/
42. poverty/
43. social class/
44. Healthcare Disparities/
45. Health Status Disparities/
46. Poverty areas/
47. Urban population/
48. (SES or SEP or sociodemographic* or "socio-demographic*" or income or wealth* or literacy or poverty or education or "educational level" or "educational attainment" or "well educated" or "better educated" or unemploy* or "home owner*" or tenure or affluen* or "well off" or "better off" or "worse off").tw.
49. (unmet adj3 need*).tw.
50. (financial adj1 (resources or situation)).tw.
51. (geograph* or regional or postcode or rural).tw.
52. (communication or information or informed or knowledge).tw.
53. Communication/
54. or/35-53
55. exp United Kingdom/
56. (national health service* or nhs*).ti,ab,in.
57. (english not ((published or publication* or translat* or written or language* or speak* or literature or citation*) adj5 english)).ti,ab.
58. (gb or "g.b." or britain* or (british* not "british columbia") or uk or "u.k." or united kingdom* or (england* not "new england") or northern ireland* or northern irish* or scotland* or scottish* or ((wales or "south wales") not "new south wales") or welsh*).ti,ab,jw,in.
59. (bath or "bath's" or ((birmingham not alabama*) or ("birmingham's" not alabama*) or bradford or "bradford's" or brighton or "brighton's" or bristol or "bristol's" or carlisle* or "carlisle's" or (cambridge not (massachusetts* or boston* or harvard*)) or ("cambridge's" not (massachusetts* or boston* or harvard*)) or (canterbury not zealand*) or ("canterbury's" not zealand*) or chelmsford or "chelmsford's" or chester or "chester's" or chichester or "chichester's" or coventry or "coventry's" or derby or "derby's" or (durham not (carolina* or nc)) or ("durham's" not (carolina* or nc)) or ely or "ely's" or exeter or "exeter's" or gloucester or "gloucester's" or hereford or "hereford's" or hull or "hull's" or lancaster or "lancaster's" or leeds* or leicester or "leicester's" or (lincoln not nebraska*) or ("lincoln's" not nebraska*) or (liverpool not (new south wales* or nsw)) or ("liverpool's" not (new south wales* or nsw)) or ((london not (ontario* or ont or toronto*)) or ("london's" not (ontario* or ont or toronto*)) or manchester or "manchester's" or (newcastle not (new south wales* or nsw)) or ("newcastle's" not (new south wales* or nsw)) or norwich or "norwich's" or nottingham or "nottingham's" or oxford or "oxford's" or peterborough or "peterborough's" or plymouth or "plymouth's" or portsmouth or "portsmouth's" or preston or "preston's" or ripon or "ripon's" or salford or "salford's" or salisbury or "salisbury's" or sheffield or "sheffield's" or southampton or "southampton's" or st albans or stoke or "stoke's" or sunderland or "sunderland's" or truro or "truro's" or wakefield or "wakefield's" or wells or westminster or "westminster's" or winchester or "winchester's" or wolverhampton or "wolverhampton's" or (worcester not (massachusetts* or boston* or harvard*)) or ("worcester's" not (massachusetts* or boston* or harvard*)) or (york not ("new york*" or ny or ontario* or ont or toronto*)) or ("york's" not ("new york*" or ny or ontario* or ont or toronto*))))).ti,ab,in.
60. (bangor or "bangor's" or cardiff or "cardiff's" or newport or "newport's" or st asaph or "st asaph's" or st davids or swansea or "swansea's").ti,ab,in.
61. (aberdeen or "aberdeen's" or dundee or "dundee's" or edinburgh or "edinburgh's" or glasgow or "glasgow's" or inverness or (perth not australia*) or ("perth's" not australia*) or stirling or "stirling's").ti,ab,in.
62. (armagh or "armagh's" or belfast or "belfast's" or lisburn or "lisburn's" or londonderry or "londonderry's" or derry or "derry's" or newry or "newry's").ti,ab,in.
63. or/55-62
64. (exp africa/ or exp americas/ or exp antarctic regions/ or exp arctic regions/ or exp asia/ or exp australia/ or exp oceania/) not (exp United Kingdom/ or europe/)
65. 63 not 64
66. 34 and 54 and 65
67. ((systematic or Cochrane or effectiveness or qualitative or mapping or overview or realist or scoping or umbrella) adj2 review*).tw.
68. (metasynthes?s or "meta synthes?s" or "meta ethnography" or "meta analys?s").tw.
69. ((integrative or integrated) adj1 review*).tw.
70. ((evidence or research) adj1 synthes?s).tw.
71. systematic review.pt.
72. meta-analysis.pt.
73. or/67-72
74. 34 and 54 and 73
75. 66 or 74
76. limit 75 to yr="2010 -Current"

Database: Social Policy and Practice
Host: Ovid
Issue: 202401
Date Searched: 6^th^ February 2024
Searcher: SB
Hits: 61
Strategy:

1. (rare adj2 (autoimmune or autosomal or blood or bone or cardia* or cardio* or childhood or chromosom* or CNV or condition* or congenital or connective or contag* or "copy number variant" or derma* or develop* or disease* or disorder* or dominant or familial or frequency or gene* or genotype or haplotype or hereditary or immun* or inflammatory or inherited or kidney or liver or mendelian or metabolic or metasta* or monogenic or muscul* or mutat* or neuro* or paediatric or pathogen or pediatric or phenotype or polygenic or recessive or respira* or skeletal or skin or tumo* or uro* or variant* or "x linked" or zoono*)).tw.
2. "highly specialised technolog*".tw.
3. (orphan adj2 (disease* or drug* or medicine* or medicinal)).tw.
4. orphanet.tw.
5. "syndrome* without a name".tw.
6. (("low frequency" or neglected) adj3 (condition* or disease or disorder*)).tw.
7. (ultraorphan or ultrarare).tw.
8. or/1-7
9. ((gene or genes or genetic* or genomic*) adj2 (condition* or disease* or disorder* or medicine or rare)).tw.
10. 8 or 9
11. ((access* or availab* or entry or referral* or pathway* or uptake or utili?ation) adj4 (care or delay* or diagnos* or healthcare or secondary or service* or specialist or support* or time)).tw.
12. (diagnos* adj4 (delay* or error* or incorrect* or missed or time or specialist* or support)).tw.
13. misdiagnos*.tw.
14. ((healthcare or "health care" or "health service*" or information* or specialist*) adj3 (need* or support*)).tw.
15. ((GP* or "general practitioner*" or primary care) adj2 referral*).tw.
16. or/11-15
17. 10 and 16
18. ((gene or genes or genetic* or genomic* or "exome sequencing") adj4 (counsel* or diagnos* or service* or test*)).tw.
19. (access* or availab* or delay* or distribution or entry or need or needs or referral* or pathway* or receiving or uptake or utili?ation).tw.
20. 18 and 19
21. 17 or 20

Web searches

Search engines

Search engine: Google Search
URL: [www.google.co.uk](http://www.google.co.uk)
Date searched: February 2024
Searcher: SB
Search terms:

#1 ("rare disease" OR “rare condition”) diagnosis (equity OR inequity) 220 hits

#2 ("rare diseases" OR “rare condition”) access (equity OR inequity) 207 hits

#3 ("rare diseases" OR “rare condition”) service (equity OR inequity) 190 hits

Websites

Organisation: Beacon
URL: <https://www.rarebeacon.org/>
Date Searched: 20^th^ February 2024
Searcher: SB
Pages browsed: <https://www.rarebeacon.org/research/>
Searches:

site:https://www.rarebeacon.org/ diagnosis 212 hits

site:https://www.rarebeacon.org/ access 97 hits

site:https://www.rarebeacon.org/ equity 6 hits

site:https://www.rarebeacon.org/ inequity 1 hit

Organisation: Organisation: Breaking Down Barriers
URL: <https://breaking-down-barriers.org.uk/>
Date searched: 20^th^ February 2024
Searcher: SB
Pages browsed:

<https://breaking-down-barriers.org.uk/research/>
<https://breaking-down-barriers.org.uk/reports/>

Searches:

site:<https://breaking-down-barriers.org.uk/> diagnosis 63 hits

site:https://breaking-down-barriers.org.uk/ access 49 hits

site:https://breaking-down-barriers.org.uk/ equity 131 hits

site:https://breaking-down-barriers.org.uk/ inequity 0 hits

Organisation: Genetic Alliance UK
URL: <https://geneticalliance.org.uk/>
Date searched: 20^th^ February 2024
Searcher: SB
Pages browsed:

<https://geneticalliance.org.uk/our-work/access-to-treatment/>
<https://geneticalliance.org.uk/our-work/diagnosis/>

Searches:

site:<https://geneticalliance.org.uk/> diagnosis 288 hits

site:<https://geneticalliance.org.uk/> access 293 hits

site:<https://geneticalliance.org.uk/> equity 57 hits

site:<https://geneticalliance.org.uk/> inequity 41 results

Organisation: Medics 4 Rare Diseases
URL: <https://www.m4rd.org/>
Date searched: 21^st^ February 2024
Searcher: SB
Pages browsed:

<https://www.m4rd.org/m4rdresources/>

Searches:

site:<https://www.m4rd.org/> diagnosis 284 hits

site:<https://www.m4rd.org/> access 131 hits

site:<https://www.m4rd.org/> equity 19 hits

site:<https://www.m4rd.org/> inequity 9 hits

Organisation: Rare Disease UK
URL: <https://geneticalliance.org.uk/campaigns-and-research/rare-disease-uk/>
Date searched: 21^st^ February 2024
Searcher: SB
Pages browsed:

https://geneticalliance.org.uk/campaigns-and-research/rare-disease-uk/

Searches:

site:<https://geneticalliance.org.uk/campaigns-and-research/rare-disease-uk/> diagnosis 0

site:<https://geneticalliance.org.uk/campaigns-and-research/rare-disease-uk/> access 0

site:<https://geneticalliance.org.uk/campaigns-and-research/rare-disease-uk/> equity 0

site:<https://geneticalliance.org.uk/campaigns-and-research/rare-disease-uk/> inequity 0

Organisation: Rare Minds
URL: <https://www.rareminds.org>
Date searched: 21^st^ February 2024
Searcher: SB
Pages browsed:

<https://www.rareminds.org/articles/>

Searches:

site:https://www.rareminds.org/ diagnosis 14 hits

site:https://www.rareminds.org/ access 28 hits

site:https://www.rareminds.org/ equity 0 hits

site:https://www.rareminds.org/ inequity 0 hits
