# Supplementary material for: Evidence of inequities experienced by the rare disease community with respect to receipt of a diagnosis and access to services: a scoping review of UK and international evidence
Source: Orphanet J Rare Dis. 2025 Jun 12;20:303. doi: 10.1186/s13023-025-03818-w (PMC12164139; doi:10.1186/s13023-025-03818-w)
Supplement: Supplementary file 2 — Additional file 2. [file 13023_2025_3818_MOESM2_ESM.docx]

Supplementary File 2. Key characteristics tables of included UK primary studies and systematic reviews

*Table S1. Key characteristics of UK primary studies*

| **Study** | **Disease(s) of interest** | **Type of study** | **Participant population, n** | **PLWRD age group (Adult/Child/Both)** | **Average age (median, range)*** | **Gender, n (%)*** |
| --- | --- | --- | --- | --- | --- | --- |
| Akanuwe 2020^33^ | Guillain-Barre Syndrome | Qualitative | PWRD (16) | Adult | NR (range 30-79 years) | Female: 7 (43.75); Male: 9 (56.25) |
| Al-Attar 2018^34^ | Tumour Necrosis Factor Receptor Associated Periodic Syndrome (TRAPS) | Qualitative (Case study) | PWRD (2) | Adult | NR | Female: 2 (100) |
| Aldiss 2021^35^ | Undiagnosed genetic condition | Qualitative | Carers (14) | Child | PLWRD: NR (range 14 months-8 years) | Female: 14 (100) |
|  |  |  |  |  | Carers: NR |  |
| Aljuburi 2012a^37^ | Sickle cell disease | Qualitative | PWRD (10) | Both | NR (range 9-56) | Female: 8 (80); Male: 2 (20) |
| Aljuburi 2012b^36^ | Sickle cell disease | Quantitative | PWRD (40) | NR | NR | Female: 20 (50); Male: 20 (50) |
| Aubeeluck 2012^38^ | Huntington disease | Qualitative | Carers (47) | NR | NR | NR |
| Bell 2021^39^ | Ehlers-Danlos syndrome and hypermobility disorders | Mixed methods | Carers (297, survey; 13, interviews) | Child | NR | *Survey*  Female: 290 (97.64); Male: 7 (2.36) |
|  |  |  |  |  |  | *Interviews*  Female: 11 (84.62); Male: 2 (15.38) |
| Bennett 2021^40^ | Ehlers–Danlos syndrome | Qualitative | PWRD (17) | Adult | NR | Female: 14 (82.35); Male 3: (17.65) |
| Berghs 2022^18^ | Sickle cell disease | Mixed methods | PWRD: (37, survey; 8, interviews) | Both | NR | *Survey* Female: 39 (76.47); Male: 11 (21.57); Other:1 (1.96) |
|  |  |  | Carers: (14, survey) |  |  | *Interviews* Female: 6 (75); Male: 2 (25) |
| Berghs 2024^41^ | Sickle cell disease (during COVID-19 pandemic) | Qualitative | PWRD (8) | Adult | NR | Female: 6 (75); Male: 2 (25) |
| Booth 2023^42^ | Inherited bleeding disorders | Quantitative | PWRD (70) | Adult | 39.9 years (mean) (SD 14.74, range 20-80) | Female: 8 (11.43); Male: 62 (88.57) |
| Cammidge 2016^43^ | Cystic fibrosis | Qualitative | PWRD (11) | Adult | NR (range 22-41) | Female: 11 (100) |
| Cassidy 2023^44^ | Phenylketonuria | Mixed methods^†^ | Carers (169) | Child | NR | Female: 154 (91.67); Male: 14 (8.33)^a^ |
| Chakravorty 2018^45^ | Sickle cell disease | Quantitative | PWRD (502); Carers (220) | Both | NR | NR |
| Chudleigh 2016^11^ | Rare diseases (multiple) | Qualitative | Carers (22) | Child | PWRD: 5.67 months (mean) (range 3-11 months) | NR |
|  |  |  |  |  | Carers: NR |  |
| Church Smith n.d.^46^ | Fabry diseases | Quantitative | PWRD (539) | NR | NR | NR |
| Clark 2019^19^ | Osteogenesis imperfecta | Quantitative | PWRD (92) | Child | 9 years (mean) (range 8 months-18 years) | Female: 40 (43); Male: 52 (57) |
| Cohen 2017^47^ | 22q11 deletion syndrome | Mixed methods^†^ | PWRD (1); carers (33) | Child | NR | NR |
| Collins 2023^48^ | Cystic fibrosis diabetes | Qualitative | PWRD (8) | Adult | 32.88 years (mean) | Female: 4 (50); Male: 4 (50) |
| Combs 2013^49^ | Retinal dystrophies | Qualitative | PWRD and carers (20) | Both | NR | NR |
| Costa 2022^50^ | Rare diseases (multiple) | Qualitative | *Interview* Carers (10)^‡§^ | Both | NR | *Interviews* Female: 7 (70); Male: 3 (30) |
|  |  |  | *Feedback events* PWRD (1); carers (6)^‡§^ |  |  | *Feedback events* Female: 4 (66.67); Male: 2 (33.33) (carers only reported) |
|  |  |  | *Joint event*  PWRD (1); carers (5)^‡§^ |  |  | *Joint event*  Female: 3 (60); Male: 2 (40) (carers only reported) |
|  |  |  | *Co-design meetings* PWRD (1); carers (1)^‡§^ |  |  | *Co-design meetings* Female: 1 (100) (carers only reported ) |
| Crowe 2019^51^ | Rare diseases (multiple) | Mixed methods^†^ | PWRD (75); carers (89)^‡^ | Both | NR | PLWRD and carers: Female (71); Male (29) |
| Cunniff 2015^52^ | Duchenne Muscular Dystrophy | Qualitative | Carers: (15, interviews; 55 written accounts of interview questions) | Both | PWRD: 16.1 (mean) (range 8-32) | *Interviews* Male: 15 (100) |
|  |  |  |  |  | Carers: 48.4 (mean) (range 34-60) | *Written accounts*  Male: 55 (100) |
| Daker-White 2015^54^ | Progressive ataxia | Qualitative | PWRD (38) | Adult | 50.5 (range 22-77) | Female: 18 (47.37); Male: 20 (52.63) |
| Daker-White 2013^53^ | Ataxia | Qualitative | PWRD (38) ‡ | Adult | 50.5 (range 22-77) | Female: 18 (47.37); Male: 20 (52.63) |
| Dures 2011^55^ | Epidermolysis Bullosa | Qualitative | PWRD (24) | Adult | NR (range 21-89) | Female: 18 (58.33); Male: 10 (41.67) |
| Fixter 2017^56^ | Cystic fibrosis | Qualitative | Carers (12) | Child | PLWRD: 8.1 (mean) (SD= 4.1, range 2-14) | Female: 10 (83.33); male: 2 (16.67) |
|  |  |  |  |  | Carers: 45.2 (mean) (SD=9.8, range 35-70) |  |
| Flaherty 2024^57^ | Trimethylaminuria | Quantitative | PWRD and carers (44) | Both | NR | Female: 34 (77.27); Male: 9 (20.45); Prefer not to say:1 (2.27) |
| Franklish 2022^58^ | Rare diseases (multiple) | Qualitative | PWRD and carers (NR) | Both | NR | NR |
| Genetic Alliance 2023^59^ | Rare diseases (multiple) | Qualitative (Case studies) | PWRD (6); Carers (3)^‡^ | Both | NR | *PLWRD* Female: 3 (50); Male: 3 (50) |
|  |  |  |  |  |  | *Carers* Female: 2 (66.67); Male: 1 (33.33) |
| Gilfillan 2024^60^ | Mayer-Rokitansky-Kuster-Hauser syndrome | Qualitative | PWRD (13) | Adult | 38.54 (mean) (range 18-59) | Female: 13 (100) |
| Gleeson 2013^61^ | Congenital adrenal hyperplasia | Quantitative | PWRD (53) | Both (transition from child to adult care) | 25.5 (range 18.4-47.8) | Female: 30 (56.60); Male: 23 (43.40) |
| Griffith 2011^62^ | Rare Genetic Intellectual Disability Syndrome (multiple) | Qualitative | Carers (8) | Adult | PLWRD: 27 (range 24-44) | *PLWRD*  Female: 6 (66.67); Male: 3 (33.33) |
|  |  |  |  |  | Carers: 54.5 (range 51-72 | *Carers*  Female: 8 (100) |
| Grose 2014^63^ | Hereditary spastic paraparesis | Qualitative | PWRD (14); Carers (6)^‡^ | Adult | NR | NR |
| Gysels 2011^64^ | Motor neurone disease | Qualitative | PWRD (10)^b^ | Adult | 42 (median) (range 24-77) | Female: 1 (10); Male: 9 (90) |
| Hagena 2014^65^ | Motor Neurone Disease | Mixed methods | *Focus groups* PWRD (5); carers (8); | NR | PLWRD: 67.64 (mean) (range 46-84) | *PLWRD* Female: 9 (81.82); Male: 2 (18.18) |
|  |  |  | *Interviews* PWRD (6); carers (4) |  | Carers: NR | *Carers*  Female: 6 (50); Male: 6 (50) |
|  |  |  | *Questionnaire* PWRD (19)^‡^ |  |  |  |
| Haig-Ferguson 2023^66^ | Oesophageal atresia and tracheo-oesophageal fistula | Qualitative | PWRD (16); carers (23) | Both (transition from child to adult care) | NR | *PLWRD*  Female: 14 (87.50); Male: 2 (12.50) |
|  |  |  |  |  |  | *Carers* Female: 21 (91.30); Male: 2 (8.70) |
| Harris 2018^67^ | Motor Neurone Disease | Qualitative | PWRD (4) | Adult | NR | NR |
| Hassal 2022^68^ | Lysomal acid lipase deficiency | Qualitative | Carers (8) | Child | NR (all <10) | Female: 5 (62.50); Male: 3 (37.50) |
| Hay 2022^69^ | Tuberous sclerosis complex, Bardet Biedl Syndrome, ANCA-associated Vasculitis | Quantitative | PWRD (40) | Both | NR | NR |
| Hill 2013^70^ | Motor neurone disease | Qualitative | PWRD (7); carers (7) | Adult | PLWRD: 66.4 (mean) (range 46-79) | *PLWRD* Female: 4 (57.14); Male: 3 (42.86) |
|  |  |  |  |  | Carers: 63.2 (mean) (range 45-70) | *Carers* Female: 3 (42.86); Male: 4 (57.14) |
| Husson 2019^71^ | Desmoid fibromatosis | Qualitative | PWRD: (14, focus groups; 13, interviews) | Adult | 39.5 (mean) (SD=13.7, range 23-74) | Female: 15 (55.56); Male: 12 (44.44) |
| Hytiris 2021^72^ | Rare diseases (multiple) | Quantitative | PWRD (13); carers (117) | Child | NR | NR |
| Iles 2010^73^ | Cystic fibrosis | Qualitative | PWRD (32)^c‡^ | Both | 19.1 (mean) | Female: 17 (53.13); Male: 15 (46.88) |
| Kalsi 2012^74^ | Inherited bleeding disorders | Quantitative | PWRD (105)^‡^ | Both | NR | NR |
| Khair 2019^76^ | Inherited bleeding disorders | Mixed methods ^†^ | Carers (231) | Child | NR | Female: 131 (56.96); Male: 99 (43.04)^d^ |
| Khair 2022^17^ | Inherited bleeding disorders | Mixed methods | PWRD and carers: (280, survey; 11 focus group; 2, interview) | Both | NR | *Survey* Female: 280 (100) |
|  |  |  |  |  |  | *Focus groups and interviews* Women: 13 (100) |
| Khair 2013^75^ | Bleeding disorders | Mixed methods | PWRD: (45, survey; NR, focus group) | Both | Survey: 19.8 (mean) (SD= 5.21, range 9-34) | *Survey* Female: 45 (100) |
|  |  |  |  |  |  | *Focus groups* Female: (100) |
| McDonald 2019^78^ | Tuberous sclerosis complex | Qualitative | PWRD (2); carers (11) | Both | NR | *PLWRD*  Female: 1(50), NR: 1 (50) |
|  |  |  |  |  |  | *Carers* Female: 4 (66.67); Male:2 (33.33); NR: 5 |
| McInnes-Dean 2024^79^ | Genetic diseases (unspecified) | Qualitative | Carers (48) ‡ | Child | Carers: 34.5 (mean) (range 28-49) | *Carers*  *Female: 42 (885); Male: 6 (13%)* |
| McMullan 2022^80^ | Rare diseases (multiple) | Mixed methods | Carers: (57, survey; 32, workshop) | Both | NR | *Survey* Female: 48 (84.21); Male: 9 (15.79) |
| Miles 2019^81^ | Sickle Cell Disease | Qualitative | PWRD (48) | Both | NR (range 13-21) | Female: 30 (62.50);  Male: 18 (37.50); |
| Morgan 2021^82^ | Dystonia | Qualitative | PWRD (8) | Adult | 52.13 (mean) (range 31-66) | Female: 5 (62.50); Male: 3 (37.50) |
| Morris 2023^16^ | Ataxia | Quantitative | PWRD (181)^§^ | Both | NR | NR |
| Morris 2022^15^ | Rare diseases (multiple) | Mixed methods | *Interviews*  PWRD (7); carers (8) | Both | NR | *PLWRD* Female: 434 (85.27); Male: 73 (14.34); Other: 2 (0.39)^e^ |
|  |  |  | *Survey* PWRD (760); carers (446)^‡^ |  |  | *Carers* Female: 235 (87.69);  Male: 32 (11.94); Other: 1 (0.37)^e^ |
| MSA Trust 2019a^83^ | Multiple system atrophy | Quantitative | PWRD and carers (284) | Adult | NR | *PWRD and carers:* Female: 122 (47%); Male: 138 (53%) |
| MSA Trust 2019b^84^ | Multiple system atrophy | Quantitative | Carers (371) | Adult | NR | Female (71); Male (28) |
| MSA Trust 2022^85^ | Multiple system atrophy | Mixed methods^†^ | PWRD (215)^f^; Carers (305) | Adult | NR | *PWRD:*  Female: 108 (51); Male: 104 (49) |
|  |  |  |  |  |  | *Carers:*  Current carers- Female: 127 (60); Male: 83 (40)  Former carers- Female: 56 (62); Male: 34 (38) |
| Neelamekam 2017^86^ | Lipoprotein lipase deficiency | Mixed methods | PWRD (3); carers (2) | Adult | PLWRD: 44.67 (mean) (range 28-64) | *PLWRD* Female: 2 (66.67); Male: 1 (33.33) |
|  |  |  |  |  | Carers: NR |  |
| O’Brien 2011a^91^ | Motor neurone disease/Amyotrophic lateral sclerosis | Qualitative | PWRD (24); carers (28) | Adult | NR | NR |
| O’Brien 2011b^88^ | Motor neurone disease/Amyotrophic Lateral Sclerosis | Qualitative | PWRD (24); carers (28) | Adult | NR | *PLWRD* Female: 16 (64); Male: 9 (36)^f^ |
|  |  |  |  |  |  | *Carers* NR |
| O’Brien 2012a^89^ | Motor neurone disease/Amyotrophic lateral sclerosis | Qualitative | Carers (28) | Adult | NR | *Carers* Male: 14 (50); Female: 14 (50) |
| O’Brien 2012b^90^ | Motor neurone disease/Amyotrophic Lateral Sclerosis | Mixed methods | *Quantitative* PWRD: (97) | Adult | *Quantitative* Females: 65.84 (mean) (SD=10.95); Males: 64.24 (mean) (SD=10.35); | *Quantitative*  Females: 48 (49.48); Males: 49 (50.51) |
|  |  |  | *Qualitative* PWRD (24); Carers (18) |  | *Qualitative* NR | *Qualitative* PLWRD: Female: 16 (64); male: 9 (36)^g^ ; Carers: NR |
| O'Brien 2015^87^ | Motor Neurone Disease | Qualitative | Carers (21) | NR | NR | Female: 10 (47.62); Male: 11 (52.38) |
| O'Brien 2023^12^ | Motor neurone disease | Mixed methods^†^ | PWRD (69); Carers (39) | Adult | PLWRD: 61 (mean) (SD=10.22, range 32-89) | *PLWRD* Female: 27 (39.71); Male: 41 (60.29) ^h^ |
|  |  |  |  |  | Carers-55 (mean) (SD=12.83, range 29-80) | *Carers* Female: 28 (77.78); Male: 8 (22.22)^h^ |
| Oerton 2011^92^ | Medium chain acyl-CoA dehydrogenase deficiency | Quantitative | PWRD (190)^i^ | Child | 9.7 days (mean) | Female: 90 (47.62%); Male: 99 (52.38%); Missing:1 |
| Pak 2020^93^ | Acromegaly | Qualitative | PWRD (18) | Adult | 52 (mean) (range 30–72) | Female: 11 (61.11); Male: 7 (38.89) |
| Pavey 2013^94^ | Motor neurone disease | Qualitative | PWRD (42) | Adult | NR | Male: 31 (73.81); Female: 11 (26.19) |
| Peter 2022^95^ | Rare diseases (multiple) | Mixed methods | *Quantitative*  PWRD and Carers (77) | Both | NR | *NR* |
|  |  |  | *Qualitative*  *PWRD and Carers (39)* |  |  |  |
| Peter 2024^96^ | Genetic diseases | Qualitative | Carers (48) ‡ | Child | Carers-34.5 (mean) (range 28-49) | *Carers*  Female: 42 (88%); Male: 6 (13%) |
|  |  |  |  |  | PWRD:NR |  |
| Pinto 2021^97^ | Motor neurone disease | Qualitative | PWRD (25); Carers (10) | Adult | NR | *PLWRD* Female: 10 (40); male: 15 (60) |
|  |  |  |  |  |  | *Carers* Female: 5 (50); male: 5 (50) |
| Limb 2010 (Rare Disease UK)^77^ | Rare diseases (multiple) | Mixed methods | PWRD (268); Carers (279) ‡ | NR | NR | NR |
| Muir 2016 (Rare Disease UK)^3^ | Rare diseases (multiple: >450) | Mixed methods | PWRD and carers (1203) | Both | NR | NR |
| Renedo 2019^98^ | Sickle Cell Disease | Qualitative | PWRD (48) | Both (transition from child to adult care) | 16.6 (mean) (range 13-21) | Female: 30 (62.50); male: 18(37.50) |
| Rodger 2015^20^ | Duchenne muscular dystrophy | Quantitative | PWRD and carers (226)§ | Both | PLWRD: 24.1 (mean) | NR |
|  |  |  |  |  | Carers: NR |  |
| Shah 2014^99^ | Rare neurodegenerative conditions | Qualitative | PWRD (15); carers (11) | Adult | PLWRD: 51.82 (mean) (range 27-79) | NR |
|  |  |  |  |  | Carers: NR |  |
| Sharma 2020^100^ | Cystic Fibrosis | Qualitative | PWRD (16) | Adult | 48 (mean) (range 24-69) | Female: 10 (62.50); Male :6 (37.50) |
| Simpson 2018^102^ | Adrenal Insufficiency/ congenital adrenal hyperplasia | Mixed methods | Carers: (20, qualitative; 57, quantitative) | Child | PLWRD: NR (range 3 months-10 years) | *Qualitative* Female: 14 (70); Male: 6 (30) |
|  |  |  |  |  | Carers: NR | *Quantitative* Female: 43 (75); Male: 14 (25) |
| Simpson 2021^101^ | Rare diseases (multiple) | Qualitative | PWRD (7); Carers (8) | Both | NR | NR |
| Skirton 2010^103^ | Huntington disease | Quantitative | Carers (108)^§^ | Adult | 53.66 (mean) (SD=12.66) | Female: 69 (63.89); Male: 39 (36.11) |
| Skrobanski 2023^104^ | Tuberous sclerosis complex | Mixed-methods ^†^ | Carers (59) | Both | 20.0 (mean) (SD=13.5) | Female: 56 (95) |
| Song 2024^105^ | Fibrous dysplasia / McCune albright syndrome | Quantitative | PWRD (51) | Adult | 51.0 (Interquartile  range: 34.5–57.5) | Female: 36 (70.59) |
| Spencer-Tansley 2018^106^ | Rare diseases (multiple) | Qualitative | PWRD and siblings (>60) | Child | NR | NR |
| Spencer-Tansley 2022^107^ | Rare diseases (multiple) | Quantitative | PWRD (913); Carers (340) | Both | NR | *Carers* Female: 315 (92.38)^j^ |
|  |  |  |  |  |  | *PLWRD* Female: 778 (83.93)^j^ |
| Specialised Healthcare Alliance 2023^24^ | Rare diseases (multiple) | Qualitative | Patients (NR); Carers (NR) ‡ | NR | NR | NR |
| Taylor 2014^108^ | Motor neurone disease | Qualitative | PWRD (13); carers (10)^k^ | Adult | PLWRD: NR (Range 43-82) | PLWRD - male 8 (61.54); female: 5 (38.46) |
|  |  |  |  |  | Carers: NR (Range 32-76 years) | Carers- male: 5 (50); female: 5 (50) |
| Trimmer 2024^109^ | Duchenne muscular dystrophy | Mixed methods | *Focus groups* PWRD (8); carers (14) | Child | NR | *Focus groups* PLWRD: Male: 8 (100) |
|  |  |  |  |  |  | *Focus groups* Carers: Female: 13 (92.86); Male: 1 (7.14) |
|  |  |  | *Survey* PWRD (18); carers (16) |  |  | *Survey*  NR |
| Twigg 2021^110^ | Polyneuropathy Organomegaly Endocrinopathy Monoclonal gammopathy Skin changes (POEMS) syndrome | Mixed methods (case study) | PWRD (1); Carer (1) | Adult | PLWRD (71) | *PLWRD* Female: 1 (100) |
|  |  |  |  |  |  | *Carer* Male:1 (100) |
| Vallortigara 2023^14^ | Progressive ataxias | Quantitative | PWRD (234); carers (39)^§^ | Both | NR | *PLWRD and carers* Female: 142 (52.59); Male: 128 (47.41) |
| Vasillca 2021^111^ | IgA Nephropathy | Qualitative | PWRD and carers (498) | NR | NR | NR |
| Walton 2023^13^ | Rare diseases | Quantitative | PLWRD (760); carers (446)^‡^ | Adult | NR | *PLWRD* Female: 434 (85.27); Male: 73 (14.34); Other: 2 (0.39)^e^ |
|  |  |  |  |  |  | *Carers* Female: 235 (87.69); Male: 32 (11.94); Other: 1 (0.37)^e^ |
| Whitaker 2021^112^ | Haemophilia A or B | Mixed methods | PWRD: (9, focus groups; 7, interviews; 226, survey) | Adult | Focus groups and interviews: 41 (median) (range 26–62) | *Focus groups* Female: 16 (100) |
|  |  |  |  |  |  | *Interviews* Female: 7 (100) |
|  |  |  |  |  | Survey: NR | *Survey* Female: 226 (100) |
| Whitehead 2012^113^ | Motor neurone disease | Qualitative | PWRD (24); carers (28) | Adult | NR | *Carers* Female:14(50); Male:14(50) |
|  |  |  |  |  |  | *PLWRD* NR |
| Wilson 2019^114^ | Cystic Fibrosis | Mixed methods | PWRD (21) | Adult | 30.9 (mean) (SD=9.1, range 18-55) | Female: 8 (38.10); Male: 13 (61.90) |
| Wray 2021^115^ | Long-segment congenital tracheal stenosis | Mixed methods | Carers (14) | Child | NR | Female: 11 (78.57); Male: 3 (21.43) |
| Wright 2022^l116^ | Previously undiagnosed developmental disorders (multiple) | Quantitative | PWRD and carers (13,450)^¶^ | Both | PLWRD: 7 (median) (range 0-63) | *PLWRD* Female (42); Male (58) |
|  |  |  |  |  | Carers: 31 (median) (range 15-90 at proband's birth) |  |
| Abbreviations: PWRD= people with a rare disease; NR= not reported  Key: *=For studies where participant population are carers only, average age and gender distribution are sometimes also reported for PLWRD who are cared for; †=survey studies which include both closed and open-ended questions and therefore classified as mixed methods; ‡=studies include service provider perspectives as well, but these are not reported here; §=studies including non-UK population samples as well, but only characteristics of UK population samples reported here; ¶=includes both UK and Ireland participants.  Footnotes: a= values calculated from 168 rather than 169 (sample total) due to missing data ; b=study included non-rare disease population sample as well, but only characteristics of rare disease population sample reported here; c=50 young people were interviewed, but only 32 young people who experienced transition and/or adult CF services are the focus of this study, and only their characteristics reported; d=values calculated from 230 rather than 231 (sample total) due to missing data; e=gender reported only for survey participants; values calculated from subsample of participant population due to missing data; f= For the PWRD group, some responses were filled in by family members, friends or healthcare professionals on behalf of the PWRD; g=values calculated from 25, rather than 24 (sample total) due to missing data; h=calculated from 68, rather than 69 (patient sample total) and 36 rather than 39 ( carer sample total) due to missing data; i=1.5 million newborns were screened, but only 190 are the focus of this study as they were presumed positive and referred for further diagnostic testing; j=calculated from 341, rather than 340 (carer total sample) and 927 rather than 913 (patient total sample) due to missing data; k=partners have been classified as carers in this study; l=in some cases both parents and offspring were analysed as a single patient unit during genomic analyses, but data reported here is divided into PLWRD (offspring) and carers (parents) for clarity. | | | | | | |

Table S2. Key characteristics of included systematic reviews

| **Study** | **Disease(s) of interest** | **Included studies, n** | **Type of included study (quant/qual/ mixed methods)** | **Countries/regions where studies carried out** | **Participant population** | **Patient age group (Adult/child/both)** |
| --- | --- | --- | --- | --- | --- | --- |
| Anderson 2021^117^ | Hypermobile Ehlers–Danlos syndrome^*^ | 13 | Qualitative; mixed methods | Belgium, Norway, Spain UK, USA | PWRD; carers^†^ | Both |
| Anestis 2020^118^ | Rare disease (multiple) | 47 | All | Australia, Canada, Europe, Germany, Greece, Italy, Netherlands, New Zealand, Norway, South Africa, Sweden, UK, USA | PWRD^†^ | Adult |
| Aoun 2013^119^ | Motor neuron disease | 59 | All | Australia, Canada, Germany, Italy, Japan, Europe, Sweden, UK, USA | Carers | NR |
| Assalone 2024^120^ | Rare diseases (multiple) | 49 | Quantitative; qualitative | Argentina, Australia, Canada, China, Denmark, Germany, Italy, Jordan, Netherlands, Poland, Portugal, Spain, Sweden, Switzerland, Taiwan, Turkey, UK, USA | Carers | Child |
| Behan 2017^121^ | Primary ciliary dyskinesia | 14 | All | Belgium, Denmark, International (25 countries), Italy, UK, USA | PWRD; carers | Both |
| Benenson 2017^122^ | Sickle cell disease | 14 | Quantitative | UK, USA | PWRD | Both |
| Best 2022^123^ | Rare diseases (multiple: non-cancer related) | 20 | All | Brazil, Canada, Malaysia, Mexico, UK, USA, | PWRD; carers^†^ | Both |
| Boersema-Wijma 2023^124^ | Huntington's disease | 38 | Quantitative; qualitative | Australia, Canada, Netherlands, New Zealand, Norway, Sweden, UK, USA | PWRD; carers^†^ | Both |
| Brandt 2022^125^ | Spinal muscular atrophy | 24 | All | Australia, Canada, China, Europe, France, Germany, Italy, Netherlands, Spain, Taiwan Turkey, UK, USA | PWRD; carers | Both |
| Bulgin 2018^126^ | Sickle cell disease | 27 | All | Brazil, Jamaica, Nigeria, UK, USA, | PWRD | Both |
| Chrastina 2023^127^ | Duchenne's Muscular Dystrophy | 8 | Qualitative | Colombia, Germany, India, Norway, UK, USA | PWRD; carers | Both |
| Coyne 2017^128^ | Cystic fibrosis | 22 | Quantitative; qualitative | Australia, Canada, France, Germany, Ireland, UK, USA, | PWRD; carers | Both |
| Fairweather 2022^129^ | Cystic fibrosis | 17 | Qualitative | Brazil, Canada, Ireland, UK, USA | PWRD | Both |
| Flemming 2020^130^ | Motor neuron disease * | 41 | Qualitative | Australia, Canada, Germany, Ireland, Italy, Japan, Netherlands, Norway, Sweden, UK, USA | PWRD; carers | Adult |
| Foley 2012^132^ | Amyotrophic Lateral Sclerosis | 47 | All | Australia, Canada, Italy, Ireland, Japan, Netherlands, New Zealand, Portugal, Scotland, Sweden, UK, USA | PWRD | Both |
| Foley 2018^131^ | Amyotrophic Lateral Sclerosis | 47 | All | Australia, Canada, Denmark, Germany, Ireland, Italy, Japan, Netherlands, Norway, South Korea, Sweden, UK, USA | PWRD; carers | NR |
| Gage 2012^133^ | Cystic fibrosis | 9 | Quantitative; qualitative | Australia, Poland, Sweden, UK, USA | PWRD | NR |
| Kamga 2020^134^ | Fragile X syndrome | 12 | Qualitative | Canada, Netherlands, South Africa, USA | Carers | Child |
| Kayle 2017^135^ | Sickle cell disease | 39 | All | UK, USA | PWRD, carers^†^ | Both |
| Khan 2023^136^ | Sickle cell disease | 59 | Quantitative | Brazil, Canada, France, French Guiana, Italy, Jamaica, Nigeria, Saudi Arabia, UK, USA | PWRD; carers | Both |
| Lal 2022^137^ | Rare diseases (multiple) | 33 | Quantitative; qualitative | Australia, Canada, Colombia, Denmark, Egypt, Europe, Germany, Greece, Italy, Netherlands, Norway, Serbia, Taiwan, UK, USA | PWRD | Both |
| Lapite 2023^138^ | Sickle cell disease | 8 | Qualitative | Canada, UK, USA, | PWRD; carers | Both |
| McMullan 2022^139^ | Rare diseases (multiple) | 35 | All | Australia, Canada, Germany, Ireland, Italy, Netherlands, Spain, UK, USA | Carers | NR |
| Milo 2022^140^ | Cystic fibrosis | 13 | Qualitative; mixed methods | Australia, UK, USA | PWRD; carers | Both |
| Nevin 2023^141^ | Childhood dementias (multiple) | 19 | Quantitative; qualitative | Australia, Canada, Germany, Ireland, Japan, Netherlands, Poland, Spain, Sweden, UK, USA | Carers | Child |
| Nunnemann 2012^142^ | Frontotemporal lobar degeneration | 19 | All | NR | Carers | NR |
| Oh 2017^143^ | Amyotrophic Lateral Sclerosis/ motor neuron disease | 37 | All | Australia, Canada, England, Germany, Italy, Japan, Korea, Netherlands, Scotland, Singapore, Sweden, Turkey, UK, USA | PWRD; carers | NR |
| Poku 2018^144^ | Sickle cell disease | 40 | All | Jamaica, Middle East, Sub-Saharan Africa, UK, USA | PWRD | Both |
| Poppe 2020^145^ | Amyotrophic lateral sclerosis | 48 | Quantitative; qualitative | Australia, Canada, Germany, Ireland, Italy, Norway, South Korea, Sweden, Turkey, UK, USA | PWRD; carers | Both |
| Porteous 2021^146^ | Duchenne's Muscular Dystrophy | 21 | Qualitative | NR | Carers | Both |
| Rance 2019^147^ | Sickle cell disease | 6 | Quantitative; qualitative | Brazil, Cameroon, Jamaica, USA | PWRD | Adult |
| Rea 2021^148^ | Sickle cell disease | 44 | All | NR | PWRD; carers^a†^ | Both |
| Rodrigues 2016^149^ | Sickle cell disease | 16 | All | NR | PWRD;  carers | Child |
| Sanigorska 2022^150^ | Bleeding disorders * | 28 | Quantitative; qualitative | Brazil, Canada, Europe, France Germany, India, Netherlands, Norway, Sweden, Turkey, UK, USA | PWRD^†^ | Both |
| Somanadhan 2023^151^ | Rare diseases (multiple) | 8 | Qualitative; mixed methods | Australia, Netherlands UK, USA | PWRD | Both |
| Tschamper 2022^152^ | Rare epilepsy-related disorders and intellectual disability (multiple) | 17 | All | Australia, Brazil Canada, France, Ireland, Netherlands, UK, USA | PWRD; carers^†^ | Both |
| Tsitsani 2023^153^ | Rare diseases (multiple) | 25 | All | Australia, Canada, China, France, Germany, Ireland, Italy, Netherlands, Spain, UK, USA | PWRD; carers | Adults |
| von der Lippe 2017^154^ | Rare diseases (multiple) | 21 | Qualitative | Canada, France, Germany, Italy, Netherlands, New Zealand, Norway, Sweden, UK, USA | PWRD | Both |
| von der Lippe 2022^155^ | Rare diseases (multiple) | 33 | Qualitative | Australia, Canada, China, Denmark, Ireland, Italy, Montenegro, Netherlands, New Zealand, Norway, Spain, Sweden, Taiwan, UK, USA | Carers | Both |
| Young 2023^156^ | Amyotrophic lateral sclerosis | 44 | All | Australia, Germany, Italy, Japan, Korea, Netherlands, Singapore, Sweden, UK, USA | PWRD; carers^†^ | Adults |
| Abbreviations: PWRD=people with a rare disease; UK=United Kingdom; USA=United States of America  Key: *= includes multiple disease types/variants; †=studies including service provider perspectives as well, but these are not reported here  Footnotes: a=studies including sibling perspectives were also included in this review, but these are not reported here | | | | | | |
