# Supplementary material for: Evidence of inequities experienced by the rare disease community with respect to receipt of a diagnosis and access to services: a scoping review of UK and international evidence
Source: Orphanet J Rare Dis. 2025 Jun 12;20:303. doi: 10.1186/s13023-025-03818-w (PMC12164139; doi:10.1186/s13023-025-03818-w)
Supplement: Supplementary file 3 — Additional file 3. [file 13023_2025_3818_MOESM3_ESM.docx]

Supplementary File 3. UK primary studies and systematic reviews with multiple rare diseases

*Table S3. Primary studies and systematic reviews reporting multiple rare diseases which are not limited to subtypes or categorises of rare disease*

| **Author year** | **Study type (primary study or SR)** | **Number of rare diseases** | **Rare diseases reported** |
| --- | --- | --- | --- |
| Muir 2016 (Rare Disease UK)^3^ | Primary study | >450 | NR |
| Morris 2022^15^ | Primary study | 221^*^ | NR^‡^ |
| Limb 2010 (Rare Disease UK)^77^ | Primary study | 119^*^ | See Annex 1 of Limb 2010 for full list.^77^ |
| Hytiris 2021^72^ | Primary study | 103^*^ | See Table 2 of Hytiris 2021 for full list.^72^ |
| Tsitsani 2023^153^ | SR | 49^†^ | Achondroplasia; Aicardi syndrome; Angelman syndrome; Arginine succinic aciduria; autoimmune thrombocytopenia; Becker syndrome; Blood diseases; Chromosome 22 Ring; Congenital malformations; Cystic fibrosis; Diseases of skin and subcutaneous tissue; Diseases of the eye and its annexes; Diseases of the genitourinary system; Disorders of the nervous system; Diseases of the respiratory system; Duchenne muscular dystrophy; Fragile X syndrome; Fryns syndrome; Gastrointestinal diseases; Genetic disorders; glycogen storage disease; Goldenhar syndrome; Guillain–Barré syndrome; Haemophilia; Hirschsprung disease; Immune system disorders; infantile agranulocytosis; Langerhans cell histiocytosis; Lesch–Nyhan syndrome; Limb–girdle muscular dystrophies; Locked-in syndrome; Mastocytosis; Moebius syndrome; Mucopolysaccharidosis type III; Musculoskeletal diseases, Metabolic disorders; Metachromatic leukodystrophy; myasthenia gravis; Neurodevelopmental disorders; Patent ductus arteriosus; polysyndactyly; Prader–Willi syndrome; Rare Diseases that require mechanical ventilation; Rett syndrome; spinal muscular dystrophies; tetralogy of Fallo; Wilson’s disease; Wolf–Hirschhorn syndrome |
| Von der Lippe 2017^154^ | SR | 31 | Addison’s disease; Amyotrophic lateral sclerosis; Anorectal atresia; Arthrogryposis, multiplex congenital; Bechet’s disease; Cystic fibrosis; Duchenne muscular dystrophy; Dysmelia; Epidermolysis bullosa; Fabry disease; Fragile X syndrome; Gaucher’s disease; Haemophilia; Horton’s disease; Idiopathic pulmonary hypertension; Locked-in syndrome and a sixth syndrome; Marfan syndrome; Mastocytosis; Mixed connective tissue disease; Neurodegeneration with brain iron accumulation; Phelan-McDermid syndrome; Phenylketonuria; Poland syndrome; Pulmonary artery stenosis; Rare congenital medical diagnoses (not specified); Scleroderma; Tuberous sclerosis; Very Rare Syndrome ;Wilson’s disease; 22q11 deletion syndrome |
| Von der Lippe 2022^155^ | SR | 29^†^ | Alagille syndrome; Amelogenesis imperfecta; Angelman syndrome; Bartter syndrome; Brdet-Biedl syndrome; Congenital craniofacial anomaly; Cornelia de Lange; Cri du Chat; Epidermolysis bullosa; Haemophilia A and B; Juvenile Huntington's disease; Mucopolysaccharidosis type II/II/III/VI ; Neuroendocrine hyperplasia of infancy; Prader-Willis syndrome; Rett syndrome; Several chromosomal disorders; Sturge-Weber syndrome; Silver-Russel syndrome; Smith-Magenis syndrome; Spinal muscular atrophy I & II; Trisomy 9, 13, 18; Urea cycle disorders; 16p11.2 deletion syndrome |
| Assalone 2024^120^ | SR | 21^†^ | Achondroplasia, Angelman syndrome; Becker  Muscular Dystrophy; Congenital anomalies; Duchenne muscular dystrophy, Dysmelia, Oesophageal atresia; Haemorrhagic disorder, Juvenile idiopathic  rheumatoid arthritis; Leukodystrophies; Marfan syndrome; Metabolic disorders; Mitochondrial respiratory chain disorder; Mucopolysaccharidosis type III, Neuromuscular disease; Phenylketonuria; Prader Willi Syndrome; Rare disease; Rett syndrome; Short bowel syndrome, Spinal Muscular Atrophy Type I and II |
| McMullan 2022^139^ | SR | 19^†^ | ANCA-associated vasculitis; CDKL5 disorder; Dravet syndrome; Duchenne muscular dystrophy; Erdheim-Chester disease; Hereditary angioedema; Huntington’s Disease; Mucopolysaccharidosis; Multiple system atrophy; Neuromyelitis Optica; Parents of disabled children; Pompe disease; Progressive Supranuclear Palsy; Rare neurodevelopmental diseases; Rett syndrome; Spinal Muscular Atrophy; Rare disease; Systemic Sclerosis; Von Hippel-Lindau disease; |
| Walton 2023^13^ | Primary study | 15 | Allergic broncho pulmonary aspergillosis; Aplastic anaemia; Ataxia; Behcet's syndrome; Common variable immune deficiency; Dravet syndrome; Ehlers-Danlos syndrome; Idiopathic intercranial hypertension; Huntington's disease; IgA nephropathy; Multiple system atrophy; Rett syndrome; Sarcoidosis; Trachea oesophageal fistula; Tuberous sclerosis |
| Somanadhan 2023^151^ | SR | 6 | Rare or undiagnosed condition; Cystic Fibrosis; Diabetes; Haemophilia; Juvenile idiopathic arthritis, Nephritic syndrome; Sickle cell anaemia |
| Genetic Alliance 2023^59^ | Primary study | 6 | Alstrom syndrome; Cavernoma; Chronic intestinal pseudo-obstruction; Deletion on chromosome 4q; Ehlers-Danlos syndrome; Sickle cell disease; |
| Lal 2022^137^ | SR | 3^†^ | Juvenile idiopathic arthritis; Muscular dystrophies; Spina bifida |
| Hay 2022^69^ | Primary study | 3 | ANCA-associated Vasculitis; Bardet Biedl Syndrome; Tuberous sclerosis complex |
| Anestis 2020^118^ | SR | 2 | Motor neuron disease, Multiple sclerosis, Parkinson's disease |
| Chudleigh 2016^11^ | Primary study | 2 | Cystic fibrosis; sickle cell disease |
| Costa 2022^50^ | Primary study | NR | n/a |
| Crowe 2019^51^ | Primary study | NR | n/a |
| McMullan 2022^80^ | Primary study | NR | n/a |
| Peter 2022^95^ | Primary study | NR | n/a |
| Simpson 2021^101^ | Primary study | NR | n/a |
| Specialised Healthcare Alliance 2023^24^ | Primary study | NR | n/a |
| Spencer-Tansley 2018^106^ | Primary study | NR | n/a |
| Spencer-Tansley 2022^107^ | Primary study | NR | n/a |

Key: *=some non-rare diseases and rare cancers also included in this number; †=number includes a combination of different rare disease type subgroups as well as specific individual rare diseases; ‡Subset of diseases were reported but majority were not reported.
